# Characterization of Clostridium difficile Spores Lacking Either SpoVAC or Dipicolinic Acid Synthetase

**DOI:** 10.1128/JB.00986-15

**Published:** 2016-05-13

**Authors:** M. Lauren Donnelly, Kelly A. Fimlaid, Aimee Shen

**Affiliations:** a Department of Microbiology and Molecular Genetics, University of Vermont, Burlington, Vermont, USA; b Program in Cellular, Molecular and Biomedical Sciences, University of Vermont, Burlington, Vermont, USA

## Abstract

The spore-forming obligate anaerobe Clostridium difficile is a leading cause of antibiotic-associated diarrhea around the world. In order for C. difficile to cause infection, its metabolically dormant spores must germinate in the gastrointestinal tract. During germination, spores degrade their protective cortex peptidoglycan layers, release dipicolinic acid (DPA), and hydrate their cores. In C. difficile, cortex hydrolysis is necessary for DPA release, whereas in Bacillus subtilis, DPA release is necessary for cortex hydrolysis. Given this difference, we tested whether DPA synthesis and/or release was required for C. difficile spore germination by constructing mutations in either *spoVAC* or *dpaAB*, which encode an ion channel predicted to transport DPA into the forespore and the enzyme complex predicted to synthesize DPA, respectively. C. difficile *spoVAC* and *dpaAB* mutant spores lacked DPA but could be stably purified and were more hydrated than wild-type spores; in contrast, B. subtilis *spoVAC* and *dpaAB* mutant spores were unstable. Although C. difficile *spoVAC* and *dpaAB* mutant spores exhibited wild-type germination responses, they were more readily killed by wet heat. Cortex hydrolysis was not affected by this treatment, indicating that wet heat inhibits a stage downstream of this event. Interestingly, C. difficile *spoVAC* mutant spores were significantly more sensitive to heat treatment than *dpaAB* mutant spores, indicating that SpoVAC plays additional roles in conferring heat resistance. Taken together, our results demonstrate that SpoVAC and DPA synthetase control C. difficile spore resistance and reveal differential requirements for these proteins among the Firmicutes.

**IMPORTANCE** Clostridium difficile is a spore-forming obligate anaerobe that causes ∼500,000 infections per year in the United States. Although spore germination is essential for C. difficile to cause disease, the factors required for this process have been only partially characterized. This study describes the roles of two factors, DpaAB and SpoVAC, which control the synthesis and release of dipicolinic acid (DPA), respectively, from bacterial spores. Previous studies of these proteins in other spore-forming organisms indicated that they are differentially required for spore formation, germination, and resistance. We now show that the proteins are dispensable for C. difficile spore formation and germination but are necessary for heat resistance. Thus, our study further highlights the diverse functions of DpaAB and SpoVAC in spore-forming organisms.

## INTRODUCTION

The spore-forming bacterial pathogen Clostridium difficile is the leading cause of nosocomial diarrhea worldwide (1). Since C. difficile is an obligate anaerobe, C. difficile infections are transmitted by its aerotolerant, metabolically dormant spores (2). Spore germination is thus necessary for C. difficile to initiate infection (3), and spore formation during infection is necessary to transmit the infection (2). The spores excreted by infected patients are particularly challenging to health care-associated facilities because they are readily disseminated (4) and resist commonly used disinfectants, such as ethanol and detergents (5).

The ultrastructure of C. difficile spores resembles that of previously characterized bacterial spores (6, 7). The innermost region of a spore is known as the core, which consists of poorly hydrated cytosol containing the genome. Approximately half of the water in the core is replaced with calcium dipicolinic acid (DPA) in Bacillus sp. spores, and DPA comprises 5 to 15% of their dry weight (7). The core is protected by a thick layer of modified peptidoglycan known as the cortex, which maintains core dehydration by physically constraining its expansion. The cortex is in turn surrounded by a proteinaceous spore coat (8) and an outermost exosporium layer (6); the latter is found in many, but not all, bacterial spores (9).

Bacterial spores germinate when their germinant receptors bind small-molecule germinants and initiate a series of events that cause the spore to exit dormancy (7). In the model organism Bacillus subtilis, where these events have been most extensively characterized (10), binding of nutrient germinants to inner spore membrane germinant receptors triggers the release of protons and monovalent cations (Na^+^ and K^+^), increasing the core pH and facilitating metabolism (10). Subsequently, DPA is released from the spore, and partial hydration of the core is observed. DPA release is essential for activating the CwlJ cortex hydrolase (11), while the functionally redundant SleB cortex hydrolase is activated through an unknown mechanism (10). CwlJ and SleB collectively degrade the cortex layer, which allows core expansion, full hydration, and resumption of metabolism and macromolecular synthesis (7).

The C. difficile germination pathway differs significantly from the one defined in B. subtilis, since C. difficile lacks the inner-membrane germination receptors that are conserved in almost all spore-forming organisms (1). Instead, C. difficile uses the germinant receptor CspC to directly sense specific bile salts (3). CspC is a pseudoprotease that activates the serine protease CspB, which in turn proteolytically activates the SleC cortex hydrolase; the activated SleC degrades the cortex and allows core expansion (12, 13). While CspC activation also leads to DPA release (3), C. difficile cortex hydrolysis precedes DPA release, in contrast to that of B. subtilis (14, 15).

Since the order of germination events differs between B. subtilis and C. difficile, we wondered whether DPA synthesis and/or release was required for C. difficile spore germination. In B. subtilis, DPA is made in the mother cell (16) by DPA synthetase, which is encoded by two cistrons, *dpaA* and *dpaB*, of the *spoVF* operon (17). Mutation of *spoVF* prevents DPA synthesis, but the spores are too unstable to be purified because they lyse during purification (18). This lysis appears to be caused by spontaneous germination, since mutation of all three germinant receptor genes (*ger3*) allows *spoVF* mutant spores to be purified (18). Notably, the resulting DPA-less spores appear to have normal cortex and coat layers, as observed by transmission electron microscopy (TEM), but the core region is more hydrated than in wild-type (WT) spores (18). The DPA-less *ger3 spoVF* spores have reduced resistance to wet heat, glutaraldehyde, and hydrogen peroxide (18).

DPA enters the forespore during sporulation and exits the core during germination in a SpoVA-dependent manner (19). B. subtilis *spoVAC* is part of a seven-gene, σ^G^-regulated operon (*spoVAA*, *spoVAB*, *spoVAC*, *spoVAD*, *spoVAEb*, *spoVAEa*, and *spoVAF* [20–22]). With the exception of those of *spoVAEa* and *spoVAF*, *spoVA* gene products are essential for stable-spore formation (23). Similar to *spoVF* mutants, *spoVA* mutants prematurely lyse during sporulation unless all three functional germinant receptor genes are deleted (24). A *ger3 spoVA* mutant strain lacks DPA even though DPA is produced in the mother cell, indicating that SpoVA proteins regulate the transport of DPA into the forespore (24). Similar to *ger3 spoVF* mutant spores, *ger3 spoVA* mutant spores also have lower core densities and heat resistance (24).

Although many bacilli encode multiple SpoVA proteins, only SpoVAC, SpoVAD, and SpoVAEb are conserved in all spore-forming bacilli and clostridia (25). SpoVAC functions as a mechanosensitive channel that likely directly transports DPA into the forespore and releases it from mature spores (19, 26), while SpoVAD binds DPA and is necessary for DPA uptake during sporulation (27). SpoVAEb appears to be an integral membrane protein (22), but its function is unknown. Whether SpoVA and DPA synthetase proteins affect spore germination and/or resistance in clostridial organisms has thus far been studied only in Clostridium perfringens.

Inactivation of the *spoVA* locus (*spoVAC-spoVAD-spoVAEb*) in C. perfringens prevents DPA transport into the forespore (28), similar to B. subtilis; however, in contrast to B. subtilis, a Δ*spoVA* C. perfringens mutant still completes sporulation and produces stable spores (28). C. perfringens *ΔspoVA* spores have a higher water content than wild-type spores and are more sensitive to wet heat (28), similar to a B. subtilis *ger3 spoVA* mutant (24). C. perfringens DPA-less Δ*spoVA* spores also exhibit a minor (20-fold) defect in germination relative to the wild type (28), whereas *B. subtilis spoVA* mutants germinate spontaneously.

Although the *spoVF* locus is conserved in most clostridia, including C. difficile, class I clostridia, such as C. perfringens, lack *spoVF* orthologues (29). Instead, C. perfringens encodes EftA, an electron transfer flavoprotein that serves as an alternate and potentially more ancient DPA synthetase (29). Sporulating *etfA* mutant cells contain significantly less DPA (<11%) than wild-type cells and are blocked at a late stage of sporulation (29). The endospores produced by this strain initially appear phase bright, but once the mother cell lyses, the spores are unstable (29), similar to *B. subtilis spoVF* mutants. While the sporulation defect of B. subtilis *spoVF* cells can be rescued by supplying exogenous DPA to permit stable-spore formation (18), exogenous DPA fails to rescue sporulation in a C. perfringens *etfA* mutant for unknown reasons (29).

In this study, we used targeted mutagenesis to investigate whether the highly conserved DpaAB and SpoVAC sporulation-specific proteins (25) regulate C. difficile spore formation, germination, and/or resistance. Functional analyses of *dpaAB* mutants revealed that DPA synthesis is dispensable for C. difficile spore formation, in contrast to B. subtilis and C. perfringens. C. difficile SpoVAC was also dispensable for spore formation and germination, in contrast to B. subtilis but largely similar to C. perfringens. Nevertheless, the DPA-less C. difficile *dpaAB* and *spoVAC* mutant spores were more susceptible to wet heat, as observed in B. subitlis and C. perfringens. These analyses further highlight the differential requirements for DPA synthetase and SpoVAC during sporulation and germination among the Firmicutes.

## MATERIALS AND METHODS

### Bacterial strains and growth conditions.

C. difficile strains are listed in Table 1 and derive from the parent strain JIR8094, an erythromycin-sensitive derivative of the sequenced clinical isolate 630 (30). C. difficile strains were grown on solid BHIS medium (31) supplemented with taurocholate (TA) (0.1% [wt/vol]), thiamphenicol (5 to 10 μg/ml), kanamycin (50 μg/ml), cefoxitin (16 μg/ml), FeSO_4_ (50 μM), and/or erythromycin (10 μg/ml), as indicated. Cultures were grown at 37°C under anaerobic conditions using a gas mixture containing 85% N_2_, 5% CO_2_, and 10% H_2_. Escherichia coli strain HB101/pRK24 was used for conjugations, and strain BL21(DE3) was used for protein production. The E. coli strains (Table 1) were routinely grown in Luria-Bertani broth (LB) at 37°C with shaking at 225 rpm. The media were supplemented with chloramphenicol (20 μg/ml), ampicillin (50 μg/ml), or kanamycin (30 μg/ml), as indicated.

**TABLE 1:** Strains and plasmids used in this study

Strain or plasmid name	Strain no.	Relevant genotype or features^*a*^	Source or reference
Strains			
C. difficile			
JIR8094	11	Erythromycin-sensitive derivative of 630	55
*spo0A* mutant	35	JIR8094 *spo0A*::*ermB*	33
*sleC* mutant	47	JIR8094 *sleC*::*ermB*	12
spoVAC* strain	619	JIR8094 *spoVAC*::*ermB*	This study
*dpaAB* mutant	746	JIR8094 *dpaA*::*ermB*	This study
JIR8094/EV	72	JIR8094 carrying pMTL84151 empty vector	This study
*sleC* mutant/EV	778	JIR8094 *sleC*::*ermB*/pMTL84151	This study
spoVAC* strain/EV	771	JIR8094 *spoVAC*::*ermB*/pMTL84151	This study
spoVAC* strain/*spoVAC*	765	JIR8094 *spoVAC*::*ermB*/pMTL84151-*spoVAC*	This study
*dpaAB* mutant/EV	781	JIR8094 *dpaAB*::*ermB*/pMTL84151	This study
*dpaAB* mutant/*dpaAB*	785	JIR8094 *dpaAB*::*ermB*/pMTL84151-*dpaAB*	This study
E. coli			
DH5α	41	λ^−^ ϕ80d*lacZ*ΔM15 Δ(*lacZYA-argF*)*U169 recA1 endA1 hsdR17*(r_K_^−^ m_K_^−^) *supE44 thi-1 gyrA relA1*	D. Cameron
	1525	pET22b-*dpaA*	This study
HB101/pRK24	531	F^−^ *mcrB mrr hsdS20*(r_B_^−^ m_B_^−^) *recA13 leuB6 ara-13 proA2 lavYI galK2 xyl-6 mtl-1 rpsL20*/pRK24	C. Ellermeier, University of Iowa
DH5α/pJS107	556	pJS107 in DH5α	3
pMTL84151 strain	703	pMTL84151 in HB101/pK424	12
BL21(DE3)	892	F^−^ *ompT hsdS*_B_(r_B_^−^ m_B_^−^) *gal dcm* (DE3)	Novagen
	1530	pET22b-*dpaA*	This study
pJS107/spoVAC* strain	1428	pJS107-*spoVAC* targeting bp 177 in HB101/pK424	This study
pJS107/*dpaAB* strain	1517	pJS107-*dpaA* targeting bp 666 in HB101/pK424	This study
pMTL84151-*spoVAC* strain	1549	pMTL84151-*spoVAC* in HB101/pK424	This study
pMTL84151-*dpaAB* strain	1590	pMTL84151-*dpaAB* in HB101/pK424	This study
Plasmids			
pMTL84151		Multicopy complementation plasmid; Cam^r^	56
pET22b		IPTG-inducible expression plasmid	Novagen
pJS107		TargeTron construct based on pJIR750ai (group II intron *ermB*::RAM *ltrA*); *catP*	3
pCE245		TargeTron construct based on pJIR750ai (group II intron *ermB*::RAM *ltrA*); *catP*	C. Ellermeier

a Cam^r^, chloramphenicol resistance.

### E. coli strain construction.

E. coli strains are listed in Table 1; all the primers are listed in Table S1 in the supplemental material. For disruption of *spoVAC* and *dpaA*, a modified plasmid containing the retargeting group II intron, pCE245 (a gift from C. Ellermeier, University of Iowa), was used as the template. Primers for amplifying the targeting sequence from the template carried flanking regions specific for each gene target: *spoVAC* (numbers 1714, 1715, 1716, and 532), the EBS Universal primer (Sigma-Aldrich), and *dpaA* (numbers 1814, 1815, 1816, and 532). The resulting retargeting sequences were digested with BsrGI and HindIII and cloned into pJS107 (3), which is a derivative of pJIR750ai (Sigma-Aldrich). The ligations were transformed into DH5α and confirmed by sequencing. The resulting plasmids were used to transform HB101/pRK24.

To construct the *spoVAC* complementation construct, primers 1855 and 1856 were used to amplify the *spoVAC* gene containing 97 bp upstream of *spoVAC*, using 630 genomic DNA as the template. To construct the *dpaAB* complementation construct, primers 1891 and 1892 were used to amplify the *dpaAB* operon containing 373 bp upstream of *dpaA*, using 630 genomic DNA as the template. Both complementation constructs were digested with NotI and XhoI and ligated into pMTL84151 digested with the same enzymes.

To construct a strain producing DpaA for antibody production, primer pair 1842 and 1843 was used to amplify *dpaA* using genomic DNA as the template. The resulting PCR products were digested with NdeI and XhoI, ligated to pET22b, and transformed into E. coli strain DH5α. The resulting pET22b-*dpaA* plasmid was used to transform E. coli strain BL21(DE3) for protein production.

### C. difficile strain construction.

C. difficile strains were constructed using TargeTron-based gene disruption, as described previously (32, 33) (see Fig. S1 in the supplemental material). Erythromycin-resistant patches were struck out for isolation onto the same medium, and individual colonies were screened by colony PCR for a 2-kb increase in the size of *spoVAC* (primer pair 1735 and 1736) and *dpaA* (primer pair 1842 and 1843) (see Fig. S1 in the supplemental material).

### C. difficile complementation.

HB101/pRK24 donor strains carrying the appropriate complementation construct were grown in LB containing ampicillin (100 μg/ml) and chloramphenicol (20 μg/ml) at 37°C and 225 rpm under aerobic conditions for 6 h. The C. difficile spoVAC* and *dpaAB* mutant recipient strains containing group II intron disruptions (where the asterisk indicates that *spoVAD* and *spoVAEb* transcripts may be reduced in the spoVAC* mutant background) were grown anaerobically in BHIS broth at 37°C with gentle shaking for 6 h. HB101/pRK24 cultures were pelleted at 2,500 rpm for 5 min, and the supernatant was removed. The pellets were transferred to the anaerobic chamber and gently resuspended in 1.5 ml of the appropriate C. difficile culture. The resulting mixture was inoculated onto predried, prereduced BHIS agar plates as seven 100-μl spots for 12 h. All the spots were collected anaerobically and resuspended in 1 ml phosphate-buffered saline (PBS). One hundred microliters of the resulting suspension was spread onto predried, prereduced BHIS agar plates supplemented with thiamphenicol (10 μg/ml), kanamycin (50 μg/ml), and cefoxitin (10 μg/ml), with three plates per conjugation. The plates were monitored for colony growth for 24 to 72 h. Individual colonies were struck out for isolation and analyzed for complementation using a heat resistance assay to test for functional-spore formation and Western blot analysis. At least two independent clones from each complementation strain were phenotypically characterized.

### Sporulation.

C. difficile strains were grown from glycerol stocks on BHIS plates supplemented with TA (0.1% [wt/vol]) or with TA and thiamphenicol (5 μg/ml) for strains carrying pMTL84151-derived vectors. The colonies that arose were then used to inoculate 70:30 agar plates (70:30 medium [35] is 70% SMC [34] and 30% BHIS) containing 5 μg/ml thiamphenicol or 100 μg/ml dipicolinic acid (2,6-pyridinedicarboxylic acid; Acros Organics) as needed for 18 to 24 h, depending on the assay. Sporulating cells were harvested into PBS, pelleted, and resuspended in PBS for visualization by phase-contrast microscopy and further processing as needed.

### Heat resistance assay of sporulating cells.

C. difficile strains were induced to sporulate as described above for 24 h, and functional (heat-resistant)-spore formation was measured as previously described (13). Heat resistance efficiencies were determined based on the average ratio of heat-resistant cells to total cells for a given strain relative to the wild type. The results are based on a minimum of three biological replicates.

### Spore purification.

After inducing sporulation on 70:30 agar plates (with 5 μg/ml thiamphenicol when appropriate) for 2 or 3 days, spores were harvested into ice-cold water as previously described (31) with the following modifications. Eight plates were typically used to harvest spores from each strain. The spores were incubated at 4°C overnight following multiple washes with water. The following day, the spores were pelleted and treated with DNase I (New England BioLabs) at 37°C for 60 min. Following DNase treatment, the spores were purified on a Histodenz (Sigma-Aldrich) gradient (either 50% or 45% for spoVAC* and *dpaAB* mutant strains), washed in water, and evaluated for purity by phase-contrast microscopy, and the optical density at 600 nm (OD_600_) of the suspension was measured. The spores were stored in water at 4°C.

### Antibody production.

The anti-DpaA antibody used in this study was raised against DpaA-His_6_ in rabbits by Cocalico Biologicals (Reamstown, PA). The DpaA-His_6_ was purified from E. coli strain 1530 using Ni^2+^ affinity resin, as previously described (12).

### Western blot analysis.

C. difficile cell pellets were processed as previously described (33, 35). Samples were resolved by SDS-PAGE and transferred to Millipore Immobilon-FL membranes. The membranes were blocked in Odyssey blocking buffer. Rabbit polyclonal anti-DpaA antibody was used at a 1:1,000 dilution, and the anti-SleC (12) antibody was used at a 1:7,000 dilution. Polyclonal mouse anti-SpoIVA (36) was used at a 1:2,500 dilution. IRDye 680CW and 800CW infrared-dye-conjugated secondary antibodies were used at 1:20,000 dilutions. The Odyssey LiCor CLx was used to detect secondary-antibody infrared fluorescence emissions.

### Quantification of Western blots.

Quantification of anti-DpaA Western blots visualized with the Odyssey LiCor CLx was done using Image Studio version 4.0.21, with background subtraction set to “average” using top/bottom subtraction with a border width of 3 (LiCor Biosciences). Normalization was performed using the sum of all data points in each replicate, as previously described (37) (see Fig. 6 for the data, presented as an average across three biological replicates). The numerical signal for each data point in a replicate was defined as the quotient of that data point divided by the sum of all the data points in that replicate (37). The normalized value for the *dpaAB* mutant carrying the empty vector (*dpaAB* mutant/EV strain) was also subtracted from each data point within each replicate, since the strain does not produce DpaA. This value was then normalized against the value obtained for the anti-SpoIVA blot, since SpoIVA served as a loading control for the number of spores.

### Spore viability following heat treatment.

To assess the effects of incubation at different temperatures on spore viability, ∼4 × 10^7^ spores were resuspended in 400 μl of water (equivalent to 1.4 OD_600_ units), and 10 μl was serially diluted for the untreated condition. The remainder of the solution was aliquoted in 90-μl increments into four tubes, which were incubated at either 50°C, 60°C, 70°C, or 80°C for 15 min. Samples were serially diluted in PBS following heat treatment as described above. After ∼22 h, colonies arising from germinated spores were counted. *spoVAC* and *dpaA* mutant spores treated at 70°C and 80°C took ∼4 to 5 h longer to grow into countable colonies. The remaining 80 μl of the spores was pelleted and processed for Western blot analysis (33, 35).

### Total DPA quantification using *A*_270_.

To measure the total amount of DPA in the spore core based on absorbance at 270 nm (38), we modified methods used previously (3, 13). Approximately 2 × 10^7^ spores from each strain were resuspended in 1 ml of buffer 1 [0.3 mM (NH_4_)_2_SO_4_, 6.6 mM KH_2_PO_4_, 15 mM NaCl, 59.5 mM NaHCO_3_, and 35.2 mM Na_2_HPO_4_] and incubated at 37°C (background) or 100°C (total DPA) for 1 h. After incubation, samples were spun down at 15,000 rpm for 2 min; 700 μl of supernatant was transferred to UV cuvettes (BrandTech), and the *A*_270_ was determined.

### Total DPA quantification using fluorescence.

To evaluate the total amount of DPA contained within spores using terbium fluorescence (39), 10 μl of supernatant from the total DPA quantification using the *A*_270_ (see above) was added to 115 μl of buffer 2 (1 mM Tris, 150 mM NaCl) with and without 800 μM terbium chloride (Acros Organics). Samples were prepared in opaque 96-well plates (PerkinElmer) and evaluated after 15 min of incubation with terbium chloride using a Synergy H1 microplate reader (BioTek; 270-nm excitation; 420-nm cutoff; 545-nm reading; gain, 100). The reported relative fluorescence units (RFU) represent the background fluorescence (wild-type spores, without terbium, incubated at 37°C) subtracted from that of all the samples. The data represent three biological replicates.

### TEM analysis.

Approximately 1 × 10^7^ spores (0.35 OD_600_ unit) were pelleted at 15,000 rpm and resuspended in osmium tetroxide fixative for TEM analysis, and TEM grids for each sample analyzed were prepared as previously described (35). A minimum of 50 spore images chosen at random were analyzed for each strain observed. The images were measured using ImageJ. Two perpendicular core diameter measurements were taken on each image. The core diameter was defined as the distance between the outermost germ cell walls. For cortex measurements, cortex thickness was defined as the distance between the outermost germ cell wall and the cortex outer edge. To account for asymmetrical spore shapes, two orthologous cortex lengths were measured so that a minimum and a maximum cortex thickness were obtained for every spore. The minimum and maximum measurements of core diameter and cortex thickness were averaged for each spore, and the upper and lower values were discarded. The core diameter and cortex thickness reported represent the averages of these measurements.

### SleC cleavage analysis.

SleC cleavage was visualized as previously described (13), with the exception that samples diluted in BHIS were incubated at 70°C for 15 min before 90-μl aliquots were added to either water or taurocholate (1% final concentration) and then incubated for 20 min at 37°C and serially diluted as described above. The remaining samples were pelleted and prepared as described for Western blot analysis.

### RNA processing.

RNAs from WT/EV, *sleC* mutant/EV, *spo0A* mutant/EV, spoVAC*/EV, spoVAC*/*spoVAC*, *dpaAB* mutant/EV, and *dpaAB* mutant/*dpaAB* strains grown for 18 h on 70:30 sporulation medium containing thiamphenicol (5 μg/ml) were extracted for quantitative real-time PCR (qRT-PCR) analyses of the *spoVAC*, *spoVAD*, *spoVAE*, *dpaA*, and *dpaB* transcripts. The RNA was extracted using a FastRNA Pro Blue kit (MP Biomedical) and a FastPrep-24 automated homogenizer (MP Biomedical). Contaminating genomic DNA was depleted using three successive DNase treatments, and mRNA enrichment was done using an Ambion Microb*Express* bacterial mRNA enrichment kit (Invitrogen), and samples were reverse transcribed as previously described (40).

### qRT-PCR.

Transcript levels of *spoVAC*, *spoVAD*, *spoVAE*, *dpaA*, *dpaB*, and *rpoB* (housekeeping gene) were determined from cDNA templates prepared from 3 biological replicates of WT/EV, *sleC* mutant/EV, *spo0A* mutant/EV, *spoVAC* mutant/EV *spoVAC* mutant/*spoVAC*, *dpaA* mutant/EV, and *dpaA* mutant/*dpaA* strains. Gene-specific primer pairs for *spoVAC* (numbers 1735 and 1736), *spoVAD* (numbers 2030 and 2031), *spoVAEb* (numbers 1931 and 1932), *dpaA* (numbers 1820 and 1821), *dpaB* (numbers 2034 and 2035), and *rpoB* (33) were used. Quantitative real-time PCR was performed as described previously (40). Transcript levels were normalized to those of the housekeeping gene *rpoB* using the standard-curve method.

### Optical-density analysis of spore germination.

Approximately 1.5 × 10^7^ spores (0.48 OD_600_ unit) were resuspended in BHIS to a total volume of 1,100 μl. The sample was divided in two: 540 μl was added to a cuvette containing 60 μl of water, while the other sample was added to a cuvette containing 60 μl of 10% taurocholate. The samples were mixed, and the OD_600_ was measured every 3 to 6 min for 45 min.

### Terbium DPA release assay.

The amount of DPA released from spores over time was measured using a modified protocol based on that of Francis et al. (14), and ∼2.5 × 10^6^ spores were incubated in buffer 2 (1 mM Tris, 150 mM NaCl) with and without 800 μM terbium and with or without germination salts (10 mM taurocholate, 10 mM glycine) in a total volume of 125 μl. Samples were evaluated every minute, with shaking, for 2 h using a Synergy H1 microplate reader (excitation, 270 nm; emission, 545 nm; gain set to the WT sample containing no terbium or germinant). Five-minute time points were plotted until the curve plateaued. The RFU reported were derived from the terbium without germinant condition subtracted from the sample with terbium and germinant for a given strain. Each assay was performed in triplicate.

## RESULTS

### Construction of *spoVAC* and *dpaA* mutants.

**FIG 1:**
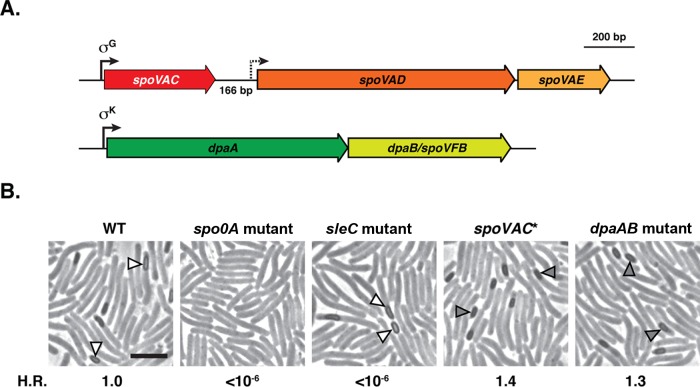
spoVAC* and *dpaAB* mutants produce phase-bright spores that are resistant to 60°C heat treatment. (A) Schematic of C. difficile *spoVAC-spoVAD-spoVAE* and *dpaAB* operons. (Top) *spoVAC* is predicted to be part of a tricistronic operon in which transcription initiates from a σ^G^-regulated promoter immediately upstream of *spoVAC*, whose position was mapped by RNA-Seq (54) (solid bent arrow). The size of the intergenic region between *spoVAC* and *spoVAD* is shown. The dashed bent arrow indicates a putative second promoter within the *spoVA* locus. (Bottom) *dpaA* is predicted to be part of a bicistronic operon that is expressed from a σ^K^-regulated promoter whose position was mapped by RNA-Seq (54) (bent arrow). (B) Phase-contrast microscopy of wild-type, *spo0A* mutant, *sleC* mutant, spoVAC*, and *dpaAB* mutant strains grown on sporulation medium for 20 h. The *spo0A* mutant cannot initiate sporulation, while the *sleC* mutant served as a control throughout the study, since it cannot germinate due to an inability to hydrolyze its cortex (14). The efficiency of heat-resistant-spore formation (H.R.) was determined for each strain relative to the wild type for three biological replicates. The white arrowheads mark phase-bright spores, while the gray arrowheads mark phase-bright spoVAC* and *dpaAB* mutant spores that are less bright than wild-type and *sleC* mutant spores. The scale bar represents 5 µm.

To determine the role(s) of SpoVAC and DpaAB in C. difficile germination, we constructed TargeTron gene disruption mutants in *spoVAC* and *dpaA* (see Fig. S1 in the supplemental material). *spoVAC* is the first gene in a potential tricistronic operon consisting of *spoVAC*, *spoVAD*, and *spoVAE*, an arrangement that is similar to that in C. perfringens (28). However, in contrast to C. perfringens, a 166-bp intergenic region separates the C. difficile *spoVAC* and *spoVAD* genes (Fig. 1). This arrangement implied that a downstream promoter might drive the expression of *spoVAD* and *spoVAE* (Fig. 1A, dashed arrow). To address this question, we analyzed the transcript reads detected during C. difficile sporulation using RNA sequencing (RNA-Seq) (40). While transcript reads were observed across the intergenic region (see Fig. S2 in the supplemental material), *spoVAD* and *spoVAEb* transcript abundance increased markedly after this region (see Fig. S2 in the supplemental material). Since these observations suggest that a downstream promoter likely also drives expression of *spoVAD* and *spoVAEb*, TargeTron insertions in *spoVAC* may not fully ablate *spoVAD* and *spoVAEb* transcription. In contrast, TargeTron insertions in *dpaA* almost certainly have polar effects on the expression of *dpaB*, since RNA-Seq analyses of the *dpaA-dpaB* region indicated that the locus forms a bicistronic operon (see Fig. S3 in the supplemental material). Based on these observations, we refer to the *spoVAC* and *dpaA* TargeTron mutants as spoVAC* and *dpaAB* mutant strains, with the asterisk indicating that *spoVAD* and *spoVAEb* transcripts may be reduced in the spoVAC* mutant background.

Analysis of spoVAC* and *dpaAB* mutants by phase-contrast microscopy revealed that both strains produced phase-bright spores, although these spores appeared less phase bright than wild-type and *sleC* mutant spores (Fig. 1B, gray versus white arrowheads). When sporulating cultures of spoVAC* and *dpaAB* mutant strains were tested for functional spore formation using an established heat resistance assay (33), both mutants produced spores that were resistant to 60°C heat treatment at levels similar to those of the wild type (Fig. 1B). These results suggest that SpoVAC and DpaAB do not affect spore formation or germination under standard laboratory conditions, unlike their equivalent mutants in B. subtilis (18, 24).

### Purification of spoVAC* and *dpaAB* mutant spores.

**FIG 2:**
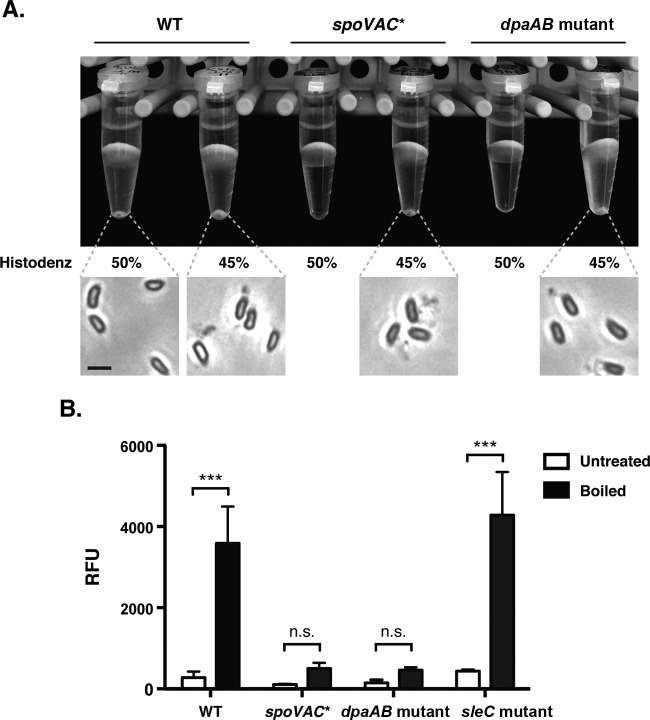
spoVAC* and *dpaAB* mutant spores appear to be less dense than WT spores. (A) Purification of wild-type, spoVAC*, and *dpaAB* mutant spores was performed using either a 50% or 45% Histodenz gradient. Visible pellets for spoVAC* and *dpaAB* mutants were detectable only using the 45% Histodenz gradient, even though similar numbers of sporulation plates were used to isolate spores for each strain and visual inspection of the samples prior to purification indicated that similar numbers of spores were produced by each strain. Representative phase-contrast microscopy images of spores isolated from the indicated strains are shown below the spore pellets analyzed. spoVAC* and *dpaAB* mutant spores largely resembled wild-type spores in size and appearance. Scale bar, 2 μm. (B) Measurement of total DPA in wild-type, *sleC* mutant, spoVAC*, and *dpaAB* mutant spores. The amount of DPA within these spores was measured using terbium fluorescence after the spores were boiled for 1 h or left untreated. The results represent the averages of three independent measurements. ***, *P* < 0.001; n.s., no statistical significance. The error bars indicate the standard deviation.

To assess whether loss of SpoVAC and/or DpaAB affected the resistance properties of C. difficile spores, as has been observed in C. perfringens (28) and B. subtilis (18, 24), we attempted to isolate spoVAC* and *dpaAB* mutant spores. Isolation of these mutant spores proved to be difficult using standard spore purification protocols (i.e., a 50% Histodenz gradient) (Fig. 2A). Reducing the density of the Histodenz gradient to 45% enabled isolation of spoVAC* and *dpaAB* mutant spores, suggesting that the mutant spores have a lower spore density than wild-type spores. This phenotype is similar to that of a C. perfringens Δ*spoVA* mutant (28) but different from the B. subtilis *spoVAC* and *dpaAB* mutants, which produce spores that are unstable unless all three germinant receptors are deleted (18, 24).

### Core density of spoVAC* and *dpaAB* mutant spores.

The apparent decrease in C. difficile spoVAC* and *dpaAB* mutant spore densities is likely due to the lack of DPA in these spores, since the equivalent mutants in B. subtilis and C. perfringens are DPA-less due to the inability of (i) *spoVA* mutants to transport DPA from the mother cell into the developing forespore (24, 28) and (ii) DPA synthetase mutants to synthesize DPA in the mother cell (17, 29). To test this hypothesis, we measured the total DPA contents of C. difficile spoVAC* and *dpaAB* mutant spores using terbium fluorescence (14, 39). The analysis revealed that spoVAC* and *dpaAB* mutant spores contain negligible amounts of DPA, in contrast to wild-type and *sleC* mutant spores (Fig. 2B). Interestingly, when total DPA levels were measured using the *A*_270_ method, spoVAC* and especially *dpaAB* mutant spores exhibited high absorbance at 270 nm, even for the untreated sample; boiling to release DPA had comparatively little impact on this value (see Table S2 in the supplemental material). The source of the intrinsic *A*_270_ signal in spoVAC* and *dpaAB* mutant spores is unknown, although it varied between spore preparations (see Table S2 in the supplemental material). Regardless, the terbium fluorescence results (Fig. 2B) indicate that the signal is not due to DPA, since Tb^3+^ specifically measures DPA levels (41), unlike the *A*_270_ measurement (28, 38, 42).

Since DPA-less B. subtilis and C. perfringens spores have increased water content (18, 24, 28), the less dense, DPA-less C. difficile spoVAC* and *dpaAB* mutant spores likely have an expanded core due to increased hydration (Fig. 2A). To test this hypothesis, we compared the core diameters of these mutant spores to that of the wild type by TEM. These analyses revealed that spoVAC* and *dpaAB* mutant spores have significantly increased core diameters compared to wild-type and *sleC* mutant spores (∼400 nm versus ∼335 nm; *P* < 0.001). We also measured the cortex thicknesses of the different strains: the cortex thickness of spoVAC* spores was similar to those of wild-type and *sleC* mutant spores (∼75 nm) (see Fig. S4 in the supplemental material), whereas the cortex of *dpaAB* mutant spores was thicker (∼85 nm; *P* < 0.001). While the significance of this difference is unclear, our results strongly suggest that spoVAC* and *dpaAB* mutant spores have increased water contents.

Since previous studies have shown that the ratio of the spore core volume to the combined volume of the cortex and core largely predicts wet-heat resistance (43), we also measured these parameters for spoVAC* and *dpaAB* mutant spores. These analyses confirmed that the core volume of these mutant spores was significantly increased (*P* < 0.0001) (see Fig. S4 in the supplemental material) relative to wild-type and *sleC* mutant spores, although no difference in the ratio of the spore core volume to the combined cortex and core volume (44) was observed between these four strains, in contrast to analyses of several different Bacillus sp. spores (43).

**FIG 3:**
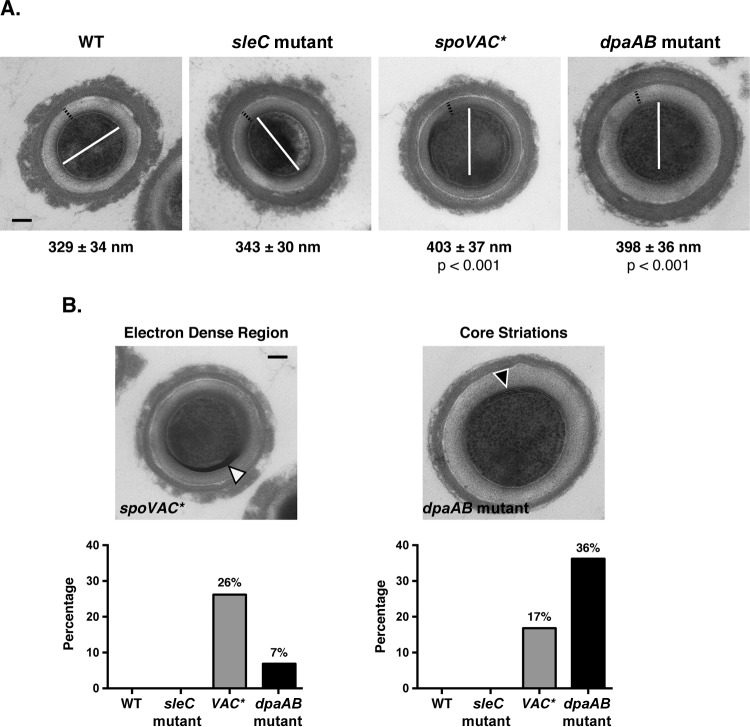
spoVAC* and *dpaAB* mutant spores have an expanded core region. (A) TEM analysis of spores. The average core diameter was determined for each sample from a minimum of 50 spores; representative images are shown. The black dotted lines represent the average cortex thicknesses measured for wild-type spores; the white solid lines represent the average core diameters determined for wild-type spores. Scale bar, 100 nm. (B) TEM analysis of spoVAC* and *dpaAB* mutant spores displaying an electron-dense region located near the germ cell wall (white arrowhead) and abnormal core striations (black arrowhead), respectively; representative images are shown. The percentages are based on >50 spores evaluated for these phenotypes for each strain. None of these features were detected in wild-type or *sleC* mutant spores. Scale bar, 100 nm.

While analyzing the TEM images, we noticed two distinct structures exclusively in DPA-less spores (Fig. 3B). First, a dark, electron-dense band between the core and germ cell wall was observed in 26% and 7% of spoVAC* and *dpaAB* mutant spores, respectively. Second, DPA-less spores contained visible striations in the core region. While electron-dense structures were more frequently observed in spoVAC* spores, core striations were more common in *dpaAB* mutant spores (17% versus 37%). These results suggest that, in addition to the apparent decrease in core density in spoVAC* and *dpaAB* mutant spores, there may be structural changes within the core and/or cortex region that may compensate for the lack of DPA in these mutant spores.

### Loss of SpoVAC and DpaAB reduces C. difficile spore heat resistance.

**FIG 4:**
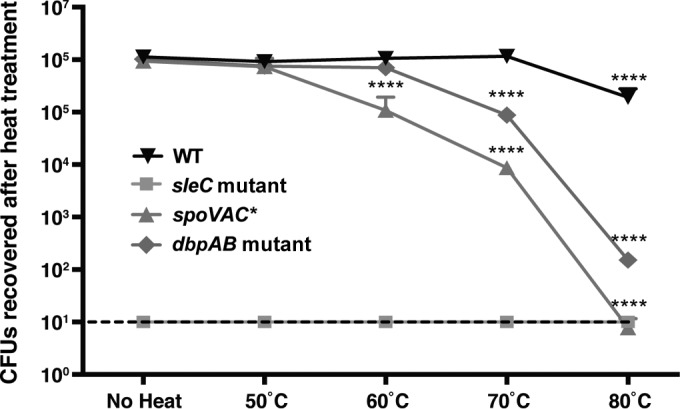
Elevated temperatures inactivate spoVAC* and *dpaAB* mutant spores (A) Wild-type, *sleC* mutant, spoVAC*, and *dpaAB* mutant spores were either left untreated or heat treated for 15 min at the indicated temperatures. The numbers of CFU produced by spores plated on BHIS containing 0.1% taurocholate are shown. The data represent averages of the results of three biological replicates. Statistical significance was evaluated using analysis of variance (ANOVA) and Bonferroni's test. The limit of detection was 10^1^ CFU. The error bars indicate the standard deviation. ****, *P* < 0.0001.

Since DPA confers heat resistance on B. subtilis and C. perfringens spores (18, 24, 28), we hypothesized that spoVAC* and *dpaAB* mutant spores would be more susceptible to wet-heat treatment at elevated temperatures. To test this, we measured the viability of spoVAC* and *dpaAB* mutant spores following exposure to increasing temperatures. Heat treatment of purified spores at 60°C for 15 min resulted in a 10-fold decrease in spore viability for the spoVAC* mutant relative to wild-type and *dpaAB* mutant spores (*P* < 0.0001) (Fig. 4). Differences in the volumes and densities of cellular materials used in this assay relative to the heat resistance assay shown in Fig. 1 may account for our inability to detect a heat resistance defect using spoVAC* sporulating cells. Interestingly, *dpaAB* mutant spore viability decreased ∼10-fold after 70°C heat treatment (*P* < 0.0001), whereas spoVAC* spore viability decreased ∼100-fold at this temperature. While heat treatment at 80°C decreased the viability of wild-type spores by ∼10-fold, spoVAC* and *dpaAB* mutant spores exhibited >5-log-unit and ∼4-log-unit defects in spore viability, respectively (Fig. 4). Taken together, these results indicate that DPA-less C. difficile spores are more susceptible to wet heat than wild-type spores, similar to B. subtilis and C. perfringens spores. Furthermore, SpoVAC appears to play additional roles in modulating C. difficile spore resistance beyond its predicted function in transporting DPA into the forespore, since C. difficile spoVAC* spores are more heat sensitive than *dpaAB* mutant spores (Fig. 4).

We next assessed whether the heat sensitivity of spoVAC* and *dpaAB* mutant spores was reversible by measuring spore viability 1, 3.5, 7, and 24 h after 80°C heat treatment. Heat-treated spoVAC* and *dpaAB* mutant spores did not recover their ability to produce CFU when plated on BHIS containing taurocholate germinant (data not shown), indicating that heat treatment irreversibly inactivates, i.e., kills these mutant spores through an unknown mechanism.

### Exogenous DPA during sporulation can complement the heat resistance defect of *dpaAB* mutant spores but not spoVAC* spores.

**FIG 5:**
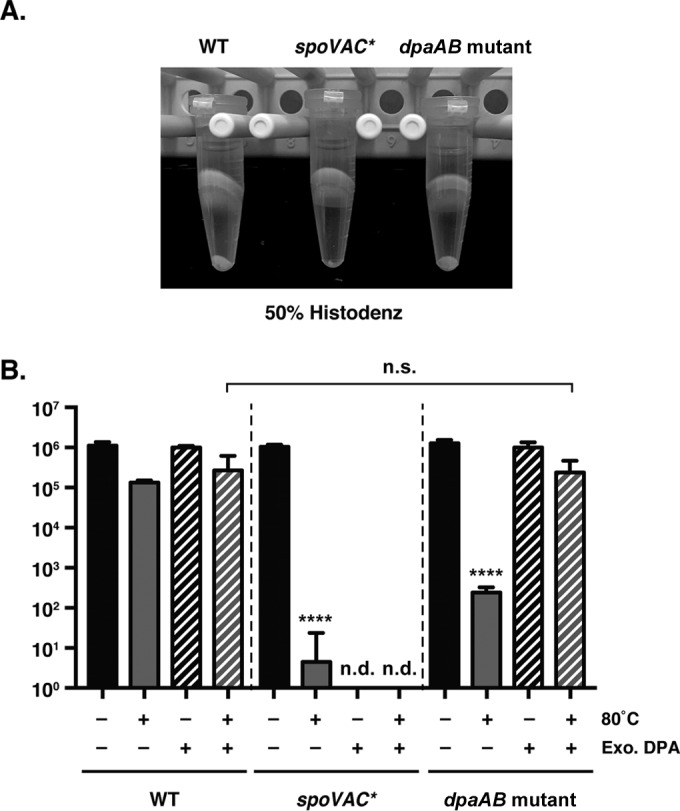
Exogenous DPA addition during sporulation rescues the wet-heat resistance of *dpaAB* mutant but not spoVAC* spores. (A) Purification of wild-type, spoVAC*, and *dpaAB* mutant spores isolated from plates containing 100 μg/ml DPA using a 50% Histodenz gradient. Spores from each strain were isolated from an equivalent number of sporulation plates, and visual inspection of the samples prior to purification indicated that similar numbers of spores were produced by each strain. (B) Heat resistance of spores produced in the presence of exogenous (Exo.) DPA. Wild-type, spoVAC*, and *dpaAB* mutant spores were produced in either the presence or absence of exogenous DPA and left untreated or heat treated for 15 min at 80°C. The numbers of CFU produced by spores plated on BHIS containing 0.1% taurocholate are shown. The data represent the averages of the results of three biological replicates. Similar results were obtained from a second set of spores purified under identical conditions. The limit of detection was 10^1^ CFU. The error bars indicate the standard deviation. ****, *P* < 0.0001; n.s., no statistical significance; n.d., not determined.

Since the sporulation defect of DPA-less *B. subtilis dpaAB*, but not *spoVA*, mutant cells can be restored by providing exogenous DPA during sporulation (24), we tested whether supplying DPA in the sporulation medium could rescue the heat sensitivity of C. difficile *dpaAB* mutant and/or *spoVAC* spores. Notably, *dpaAB* mutant, but not spoVAC*, spores produced in the presence of exogenous DPA could be efficiently purified on a 50% Histodenz gradient (Fig. 5A). Sporulation in the presence of exogenous DPA also resulted in *dpaAB* mutant spores exhibiting wild-type levels of heat resistance (Fig. 5B), although spoVAC* spores produced in the presence of exogenous DPA could not be purified at sufficient levels to test their heat resistance. Taken together, these results are consistent with a model in which *dpaAB* mutant spores take up the exogenous DPA in a SpoVAC-dependent manner, which results in increased wet core density and contributes to the heat resistance of the resulting spores.

### Complementation of spoVAC* and *dpaAB* mutants.

To confirm that the heat sensitivity of spoVAC* and *dpaAB* mutant spores was due to the absence of SpoVAC and DpaAB, we tested whether this defect could be restored by complementing the mutant strains with *spoVAC* and *dpaAB*, respectively, on a multicopy plasmid. Since Integrated Genome Browser (IGV) analyses (45) suggested that *spoVAD* and *spoVAEb* are expressed from a separate promoter downstream of the *spoVAC* promoter (see Fig. S2 in the supplemental material), we tested whether *spoVAC* alone could complement the *spoVAC* TargeTron disruption mutant. Both the *spoVAC* and *dpaAB* complementation constructs successfully restored heat resistance to wild-type levels in spoVAC* and *dpaAB* mutant spores, respectively (see Fig. 7A), indicating that the heat sensitivity of these strains was due primarily to loss of SpoVAC and DpaAB, respectively.

qRT-PCR analyses of *spoVAC*, *spoVAD*, and *spoVAEb* transcript levels in the spoVAC* strain carrying either an empty vector or the *spoVAC* complementation construct revealed that *spoVAD* and *spoVAEb* transcript levels were reduced ∼10-fold during sporulation relative to the wild type carrying the empty vector (WT/EV) (see Fig. S5 in the supplemental material). While the reduction in *spoVAEb* transcript levels in the spoVAC*/EV mutant and spoVAC*/*spoVAC* complementation strain was not statistically significant compared to that in the WT/EV strain, it approached significance (*P* < 0.11 and 0.06, respectively). Taken together, these analyses indicate that the *spoVAC* TargeTron disruption reduces but does not abolish *spoVAD* and *spoVAEb* transcription, consistent with the operon structure detected by IGV (45) analyses of our RNA-Seq data (see Fig. S2 in the supplemental material). This reduction in *spoVAD* and *spoVAEb* transcription nevertheless does not appear to contribute to the heat resistance defect of spoVAC* spores, since expression of *spoVAC* alone was sufficient to restore heat resistance to the mutant background.

**FIG 6:**
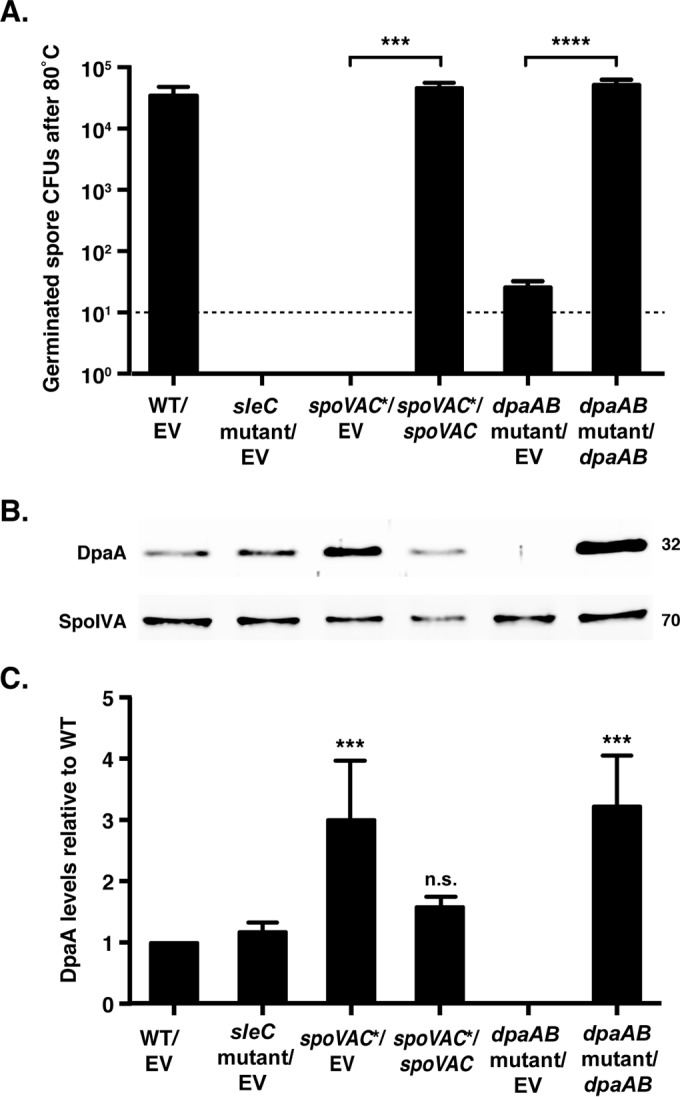
Plasmid complementation of the spoVAC* and *dpaAB* mutant strains restores wet-heat resistance. (A) Purified spores from the wild type carrying an empty vector (WT/EV), the *sleC* mutant carrying an empty vector (*sleC* mutant/EV), the spoVAC* strain carrying an empty vector (spoVAC*/EV) or the *spoVAC* complementation construct (spoVAC*/*spoVAC*), and the *dpaAB* mutant carrying an empty vector (*dpaAB* mutant/EV) or the *dpaAB* mutant complementation construct (*dpaAB* mutant/*dpaAB*) were incubated at 80°C for 15 min. The average numbers of CFU recovered on BHIS plates containing 0.1% taurocholate after heat treatment and germinant exposure across three biological replicates are shown. The limit of detection was 10^1^ CFU. Statistical significance was evaluated using ANOVA and Tukey's test (***, *P* < 0.001; ****, *P* < 0.0001). (B) Western blot analysis of spores used in the germination assay. The mouse anti-SpoIVA antibody was used as a loading control. Molecular weight markers are shown on the right. (C) Quantitation of Western blot analyses of DpaA levels in purified spores from the indicated strains. The data presented are the averages of the results from three biological replicates. Normalization was performed using the sum of all data points in each replicate, as previously described (37, 40). Similar results were obtained with spores from independent purifications. ***, *P* < 0.001; n.s., no statistical significance The error bars indicate the standard deviation.

qRT-PCR analyses of *dpaA* and *dpaB* expression confirmed that the TargeTron disruption in *dpaA* has polar effects on *dpaB* transcription (see Fig. S5 in the supplemental material) and revealed that the *dpaAB* complementation strain (*dpaAB* mutant/*dpaAB*) overexpresses both *dpaA* and *dpaB* relative to the wild type (*P* < 0.05). Western blot analyses revealed that *dpaAB* mutant/*dpaAB* spores contained ∼3-fold-higher levels of DpaA than the wild type (Fig. 6B) (*P* < 0.001). Interestingly, spoVAC* spores also exhibited a statistically significant increase in levels of DpaA (Fig. 6C), even though no difference in *dpaA* or *dpaB* expression was observed in the strain relative to the wild type (see Fig. S5 in the supplemental material). This observation suggests that DpaA is more efficiently incorporated into and/or stabilized in *spoVAC* mutant spores for unknown reasons.

### Loss of SpoVAC or DpaAB does not affect SleC cleavage.

**FIG 7:**
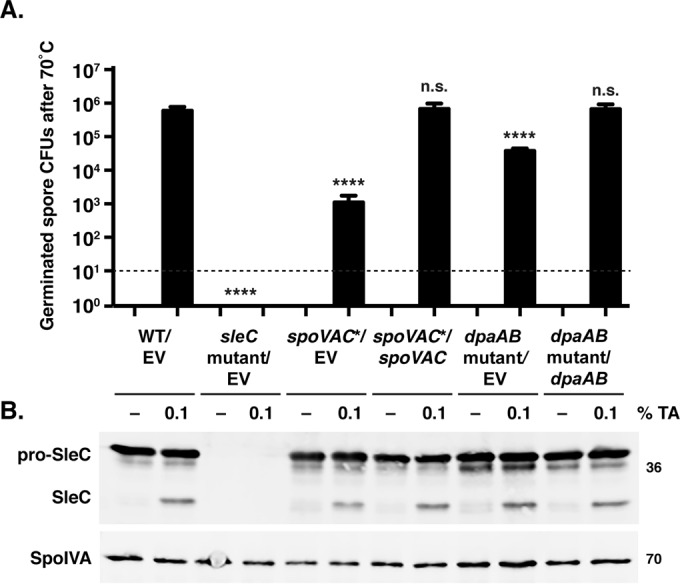
Heat treatment at 70°C does not affect SleC cleavage in spoVAC* and *dpaAB* mutant spores. (A) Germination of spores purified from the indicated strains after 70°C heat treatment for 15 min, followed by either no treatment or exposure to 1% TA. Samples were plated on BHIS. The data represent the averages of the results of three biological replicates. Statistical significance was evaluated using ANOVA and Tukey's test. ****, *P* < 0.0001; n.s., no statistical significance. (B) Western blot analyses of samples from one representative replicate of the *in vitro* germination assay. The zymogen pro-SleC undergoes CspB-dependent processing in response to germinant addition (12). The mouse anti-SpoIVA antibody was used as a loading control. The limit of detection was 10^1^ CFU. The error bars indicate the standard deviation.

Due to the irreversibility of the effect of heat treatment on spoVAC* and *dpaAB* mutant spore viability, we wondered whether germination in these mutant spores was arrested at a particular stage. We first investigated whether SleC cleavage (12) was altered in heat-treated mutant spores. Exposure of spoVAC* and *dpaAB* mutant spores to 70°C for 15 min did not affect SleC cleavage in spoVAC* and *dpaAB* mutant spores relative to the wild type when germination was induced by the addition of 0.1% TA for 20 min after heat treatment (Fig. 7B). As observed above (Fig. 4), spoVAC* and *dpaAB* mutant spores nevertheless exhibited ∼1,000- and 10-fold decreases in spore viability, respectively, relative to the wild type and their respective complementation strains when spore germination was measured by plating germinating spores on BHIS (Fig. 7A). In the absence of heat treatment, spoVAC* and *dpaAB* mutant spores also cleaved SleC and germinated at wild-type levels (see Fig. S6 in the supplemental material), confirming that loss of SpoVAC and DpaAB does not impact spore germination *per se* (Fig. 4). Interestingly, while there was no difference between SleC cleavage and spore germination in wild-type spores heated to 70°C relative to untreated spores, heating spores to 80°C prior to TA germinant addition abrogated both SleC cleavage and spore germination, even for wild-type spores (data not shown). Perhaps the 80°C heat treatment reduces the kinetics of spore germination to greater than the 20 min used in this assay, or heating spores in the presence of BHIS increases the sensitivity of even wild-type spores to 80°C. Indeed, since the germinant sensing and signaling proteins (Csps) and SleC cortex hydrolase are found outside the core region, they may be more sensitive to heat than core proteins. Regardless, our results suggest that heat treatment inactivates germination and/or outgrowth at a step downstream of germinant sensing and SleC activation.

To further test this model, we assessed whether heat-treated spoVAC* and *dpaAB* mutant spores could be recovered by a decoating and lysozyme treatment that bypasses the cortex hydrolysis step (3). Similar numbers of CFU were recovered from heat-treated (80°C) *dpaAB* mutant spores regardless of whether the spores were artificially germinated and plated on BHIS plates or naturally germinated by plating on BHIS containing taurocholate (see Fig. S7 in the supplemental material). Heat-treated spoVAC* spores were also inefficiently recovered when subjected to artificial germination (46) and plated on BHIS. Interestingly, unheated spoVAC* spores were also poorly recovered on BHIS following the artificial-germination procedure, in contrast to *sleC* mutant and *dpaAB* mutant spores, suggesting that DPA-less spoVAC* spores have structural differences from DPA-less *dpaAB* mutant spores. Regardless, these results further suggest that heat inactivates spoVAC* and *dpaAB* mutant spore germination at a stage downstream of cortex hydrolysis.

### The change in optical density of spoVAC* and *dpaAB* mutant spores during germination is diminished relative to wild-type spores.

Since SleC was cleaved in the DPA-less mutants at levels similar to those of the wild type following heat treatment, we next tested whether the decrease in OD observed during spore germination (47) was affected in heat-treated mutant spores. The drop in the OD_600_ observed during B. subtilis spore germination is due to both cortex hydrolysis and core hydration as a result of DPA release, with ∼50 to 70% of the total decrease due to DPA release (38, 48). Following germinant addition, C. difficile spoVAC* and *dpaAB* mutant spores both exhibited an ∼20% reduction in their OD_600_ after germinant addition, irrespective of heat treatment (Fig. 7 and data not shown). In contrast, wild-type spores and the spoVAC* and *dpaAB* mutant complementation strain spores exhibited an ∼40% decrease in their OD_600_ during spore germination. Since *sleC* mutant spores exhibited little change in their OD_600_ values following germinant addition and spoVAC* and *dpaAB* mutant spores cleaved SleC in response to germinant (Fig. 7), the ∼20% drop in OD_600_ observed in germinating spoVAC* and *dpaAB* mutant spores was likely due to cortex hydrolysis. The remaining ∼20% drop in the OD_600_ observed in wild-type and complementation strain spores was presumably due to core hydration upon DPA release (14), given that spoVAC* and *dpaAB* mutant spores lack DPA (Fig. 3).

**FIG 8:**
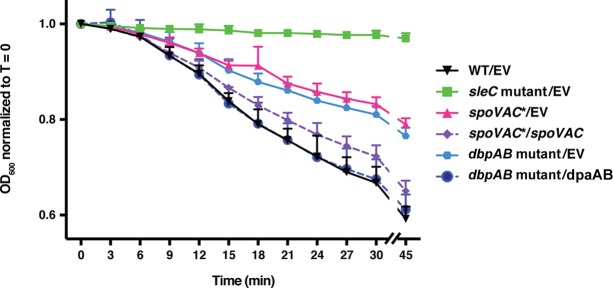
Intermediate change in the OD_600_ of spoVAC* and *dpaAB* mutant spores in response to germinant relative to wild-type spores. *sleC* mutant spores served as a negative control, since they cannot hydrolyze the cortex (14). Purified spores from the indicated strains were resuspended in BHIS, and germination was induced by adding taurocholate (1% final concentration). The ratio of the OD_600_ of each strain at a given time point relative to the OD_600_ at time zero is plotted. The averages of the results of three independent experiments are shown, and the error bars indicate the standard deviation for each time point measured.

**FIG 9:**
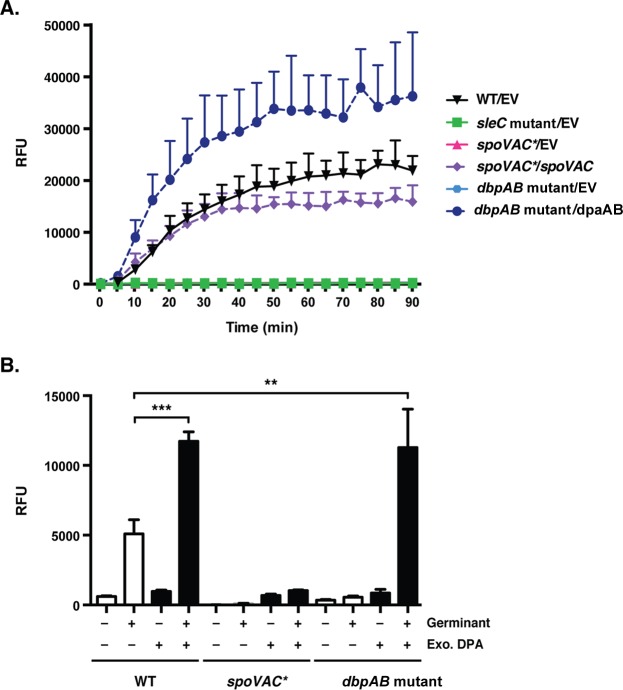
DPA release by spoVAC* and *dpaAB* mutant strains in response to germinant. (A) DPA release from germinating C. difficile spores was monitored over time using terbium fluorescence. (B) DPA released from C. difficile spores isolated in the presence or absence of exogenous (Exo.) DPA after exposure to germinant for 30 min. The data represent the average and standard deviation of the results from three biological replicates. **, *P* < 0.01; ***, *P* < 0.001. The differences between the amounts of DPA released from wild-type and *dpaAB* mutant spores produced in the presence of exogenous DPA were not significant.

To support this theory, we measured DPA release by spoVAC* and *dpaAB* mutant spores over time in response to germinant. Consistent with the negligible amounts of DPA detected in the mutant spores (Fig. 2B), spoVAC* and *dpaAB* mutants did not release DPA in response to germinant (see Fig. 9A). The *sleC* mutant (see Fig. 9A) failed to release DPA during germination, as expected (14, 15). In contrast, spoVAC*/spoVAC and *dpaAB* mutant/*dpaAB* spores released DPA with kinetics identical to those of wild-type spores, although the *dpaA* complementation strain, which contains higher levels of DpaA than the wild type (Fig. 6B), released more DPA during germination than the wild type (see Fig. 9A). Taken together, the intermediate drop in the OD_600_ during spoVAC* and *dpaAB* mutant spore germination (Fig. 8) appears to be due to the lack of DPA in these strains.

Since *dpaAB* mutant/*dpaAB* spores appeared to release ∼2-fold more DPA (Fig. 9A) and possess ∼3-fold more DPA synthetase A (Fig. 6C) than wild-type and spoVAC*/*spoVAC* spores, we wondered whether wild-type and *dpaAB* mutant spores produced in the presence of exogenous DPA might similarly take up and release more DPA than wild-type spores produced under standard conditions. Terbium fluorescence analyses revealed that ∼2-fold more DPA was released in response to germinant by wild-type and *dpaAB* mutant spores produced in the presence of exogenous DPA than by wild-type spores produced under standard conditions (Fig. 9B). Similar amounts of DPA were released by wild-type and *dpaAB* mutant spores produced in the presence of exogenous DPA, consistent with their equivalent levels of heat resistance (Fig. 5B). These results indicate that excess exogenous DPA and elevated levels of DPA synthetase can alter the amount of DPA incorporated into the spore core, but the increased DPA does not alter the extent of the OD_600_ drop during germination.

## DISCUSSION

Previous studies of B. subtilis and C. perfringens have shown that SpoVA and DPA synthetase proteins differentially regulate spore formation and germination in these organisms. In this study, we determined that DPA synthesis is dispensable for both stable-spore formation and germination in C. difficile (Fig. 2 and 4), in contrast to both B. subtilis and C. perfringens (18, 29). Nevertheless, similar to B. subtilis (24) and C. perfringens (28), C. difficile SpoVAC is necessary for transporting DPA into spores, since spoVAC* spores lack DPA (Fig. 2B), and spoVAC* spores produced in the presence of exogenous DPA did not appear to incorporate DPA into their cores, in contrast to *dpaAB* mutant spores produced in the presence of exogenous DPA (Fig. 5 and 9B). Furthermore, C. difficile SpoVAC is dispensable for stable-spore formation and germination, similar to C. perfringens (28) but in contrast to B. subtilis (24).

It is unclear why there are such different requirements for DPA synthetase and SpoVAC during spore formation and germination in C. difficile, C. perfringens, and B. subtilis. We hypothesize that these proteins are dispensable for stable C. difficile spore formation and germination because its cortex hydrolase, SleC, is not activated by DPA during germination. Although DPA activates B. subtilis spore germination (49) by activating its cortex hydrolase, CwlJ (18), DPA does not induce C. difficile spore germination (15), and its cortex hydrolase, SleC, is unaffected by DPA (15). Instead, C. difficile SleC-mediated cortex hydrolysis is essential for DPA release (14, 15).

Similar to C. difficile, C. perfringens SleC cortex hydrolase is also DPA independent (50); in contrast to C. difficile, DPA can induce C. perfringens spore germination, but only if the spores are first activated by heat. Interestingly, although we previously observed that DPA does not affect C. difficile spore germination, these analyses were not performed on heat-activated spores (15), so it is possible that DPA could induce germination of heat-activated C. difficile spores. Notably, although both C. perfringens and C. difficile SleC activity is induced by CspB-mediated proteolytic cleavage (12, 51), cortex hydrolysis accelerates but is not absolutely essential for DPA release in C. perfringens (50), in contrast to C. difficile. Our observation that the optical density at 600 nm of DPA-less spoVAC* and *dpaAB* mutant spores is reduced by ∼50% relative to wild-type spores upon germinant addition (Fig. 8) suggests that this requirement will also be observed in C. perfringens, since the OD_600_ of C. perfringens Δ*spoVA* spores decreases by ∼70% relative to that of the wild type during germination (28). While the remaining 30% drop in the OD_600_ in these Δ*spoVA* spores was attributed to an intrinsic difference in the refractility of Δ*spoVA* relative to wild-type spores (28), we obtained equivalent numbers of CFU on BHIS plates containing taurocholate for wild-type, spoVAC*, and *dpaAB* mutant spores for a given OD_600_ (Fig. 4 to 6). Taking the data together, we postulate that C. difficile and C. perfringens *spoVA* mutant spores are stable because their SleC cortex hydrolase is unaffected by DPA, in contrast to B. subtilis *spoVA* and *spoVF* mutants (18, 24).

This model nevertheless fails to explain why DPA-less C. perfringens *eftA* spores are unstable (29) relative to C. difficile *dpaAB* mutant spores, since the instability of *eftA* mutant spores is presumably independent of SleC cortex hydrolase activation. It has been proposed that the presence of SpoVA proteins in the *eftA* mutant may contribute to their instability by allowing inappropriate cation transport (29). Another possibility is that the absence of DPA synthesis in the C. perfringens mother cell leads to alterations in the cortex layer that reduce its ability to protect against osmotic stress or enhance its degradation by alternative cortex hydrolases, such as SleM (50). Consistent with the latter hypothesis, deletion of the *sleB* gene encoding a redundant cortex hydrolase that apparently becomes activated in DPA-less spores can suppress the instability of B. subtilis *spoVF* mutant spores (18). Interestingly, we observed using TEM that *dpaAB* mutant spores had a thicker cortex than wild-type, *sleC* mutant, and spoVAC* spores (Fig. 3); perhaps this thicker cortex layer is an adaptation that allows C. difficile *dpaAB* mutant spores to be stably purified.

C. difficile DPA-less spores may have additional adaptations relative to C. perfringens spores that contribute to their stability. Both spoVAC* and *dpaAB* mutant spores exhibited structural changes relative to wild-type spores (Fig. 3B), specifically, electron-dense bands and increased core striations. These morphological changes may allow the mutant spores to compensate for the change in osmolarity relative to the wild type caused by the loss of DPA. Our analyses of DPA levels using the *A*_270_ method further suggest that spoVAC* and *dpaAB* mutant spores have adapted to the absence of DPA, since these spores, especially *dpaAB* mutant spores, gave markedly higher readings than wild-type and *sleC* mutant spores even prior to boiling, at least in certain spore preparations (see Table S2 in the supplemental material). Determining the nature of these changes would provide significant insight into the mechanisms that control C. difficile spore resistance and the role of DPA in modulating this resistance.

Even though C. difficile, C. perfringens, and B. subtilis have different requirements for SpoVAC and DpaAB, these proteins control core hydration (Fig. 2 and 3) and wet-heat resistance (Fig. 4) in all three organisms. We can attribute the heat resistance defect of spoVAC* spores to the loss of *spoVAC* expression, since complementation with *spoVAC* alone restored heat resistance to wild-type levels without causing significant overexpression of *spoVAC* (Fig. 5; see Fig. S5 in the supplemental material), even though *spoVAD* and *spoVAEb* transcription was reduced ∼10-fold relative to the wild type (see Fig. S5 in the supplemental material). The presumably reduced SpoVAD and/or SpoVAEb levels in spoVAC*/spoVAC spores appear to be sufficient to carry out the normal functions of these proteins, or SpoVAD and SpoVAEb are not required for conferring heat resistance on C. difficile spores. It would be interesting to directly test the requirement for SpoVAD and/or SpoVAEb, using the recently developed allelic-exchange method (52), in future studies.

Notably, the heat sensitivity of C. difficile spoVAC* spores was 10- to 100-fold greater than that of *dpaAB* mutant spores, suggesting that SpoVAC may transport additional small molecules into the forespore and/or release other small molecules during germination that help to protect C. difficile against wet heat. In addition, the increased levels of DpaA in spoVAC* spores suggests that the spores may have a mechanism for detecting the absence of DPA and compensating for this deficiency. Since qRT-PCR analyses indicated that sporulating spoVAC* cells express *dpaAB* at levels similar to those of the wild type (see Fig. S5 in the supplemental material), spoVAC* spores appear to recruit more DpaA into a “coat-extractable” region. One possibility is that *dpaAB* mutant spores have a higher wet core density than spoVAC* spores, since the spore core water content has previously been shown to be inversely proportional to spore wet-heat resistance (43).

While the reasons for the difference in heat sensitivity between spoVAC* and *dpaAB* mutant spores remain unknown, the heat-sensitive component(s) of these DPA-less spores that limits germination and/or outgrowth also has not been identified. Wet heat has been shown to denature B. subtilis spore proteins (53), and it has been proposed that it inactivates key metabolic enzymes involved in outgrowth, since wet-heat-killed spores can still germinate but fail to grow out into rod-shaped cells (53). Consistent with that study, our analyses indicate that heat treatment does not affect SleC cleavage (Fig. 7) or cortex hydrolysis, as detected by optical-density measurements (data not shown) and artificial-germination analyses (see Fig. S7 in the supplemental material). Given that our analyses indicate that DPA does not affect either C. difficile spore formation or germination, in contrast to B. subtilis and C. perfringens, C. difficile may represent a more tractable system for identifying proteins that confer DPA-dependent wet-heat resistance. Regardless, our study indicates that there is considerable diversity in the function of DPA among the Firmicutes and provides yet another example of how conserved proteins can have different functions in C. difficile relative to other Firmicutes.

## Supplementary Material

Supplemental material
