# Supplementary material for: Characterization of Clostridium difficile Spores Lacking Either SpoVAC or Dipicolinic Acid Synthetase
Source: J Bacteriol. 2016 May 13;198(11):1694–707. doi: 10.1128/JB.00986-15 (PMC4959285; doi:10.1128/JB.00986-15)
Supplement: Supplemental material [file supp_198_11_1694__index.html]

Characterization of Clostridium difficile Spores Lacking Either SpoVAC or Dipicolinic Acid Synthetase — Supplemental material 

# Characterization of Clostridium difficile Spores Lacking Either SpoVAC or Dipicolinic Acid Synthetase

## Supplemental material

- Supplemental file 1 -

  Fig. S1, generation of mutant strains using gene disruption

  Fig. S2 and S3, visualization of RNA-seq data

  Fig. S4, transmission electron microscopy analyses of wild-type and mutant spores

  Fig. S5, qRT-PCR analyses of transcript levels

  Fig. S6, SleC cleavage and germination of untreated mutant spores

  Fig. S7, artificial germination of spores

  Table S1, primers

  Table S2, measurement of total DPA

  PDF, 3.0M
