# Supplementary material for: Characterization of Clostridium difficile Spores Lacking Either SpoVAC or Dipicolinic Acid Synthetase
Source: J Bacteriol. 2016 May 13;198(11):1694–707. doi: 10.1128/JB.00986-15 (PMC4959285; doi:10.1128/JB.00986-15)
Supplement: Supplemental material [file JB.00986-15_zjb999094055so1.pdf]

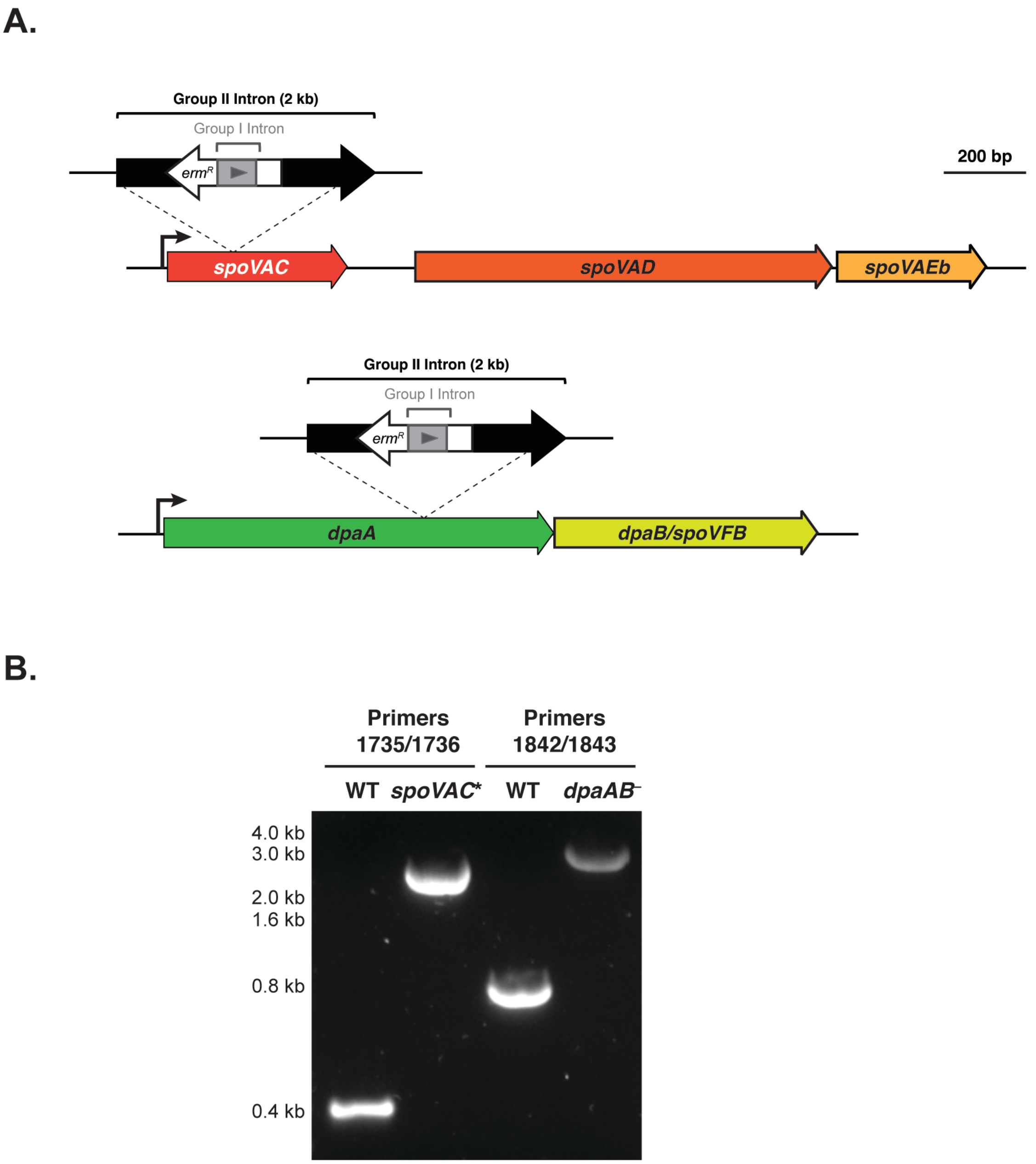

FIG S1. Generation of *spoVAC** and *dpaAB*$^{-}$ strains using Targetron gene disruption. (A) Schematic of the Group II Intron system (32) used for insertional mutagenesis of *spoVAC* (177 bp) and *dpaA* (666 bp). Bent arrows indicated the promoters mapped by RNA-Seq (51). (B)

Colony PCR of wildtype, *spoVAC**, and *dpaAB*⁻ strains using primers that flank the gene of interest. The Group II Intron is ~2 kb.

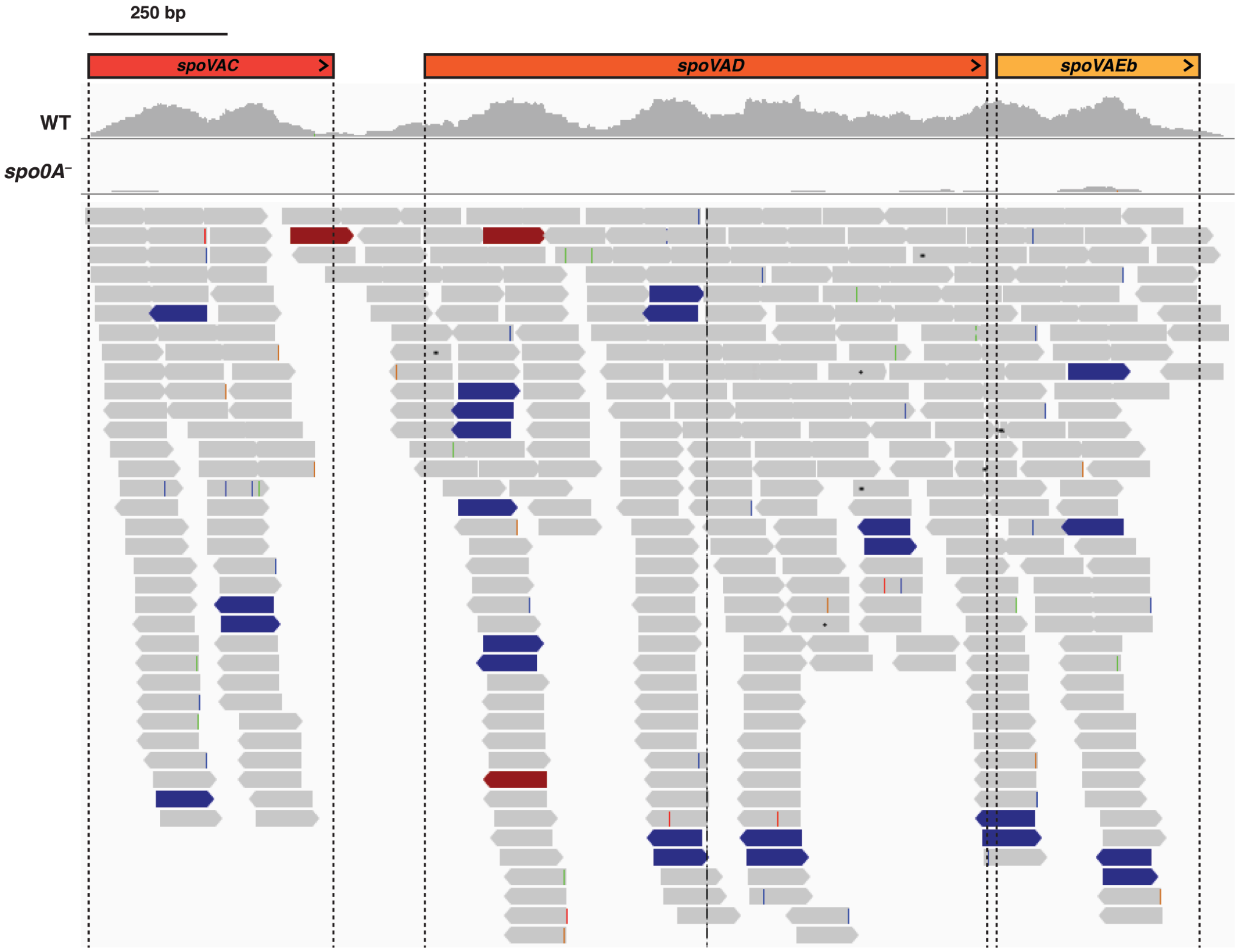

FIG S2. Representative image of Integrated Genome Viewer software (52) used to visualize of *spoVAC-VAD-VAEb* RNA-Seq data from (40). Histograms of RNA sequence reads obtained in wild type and *spo0A*⁻ are shown in grey. The direction of transcription is indicated by the angled bracket. The reads mapped to this locus are shown. A 166 bp intergenic region separates *spoVAC* and *spoVAD* genes.

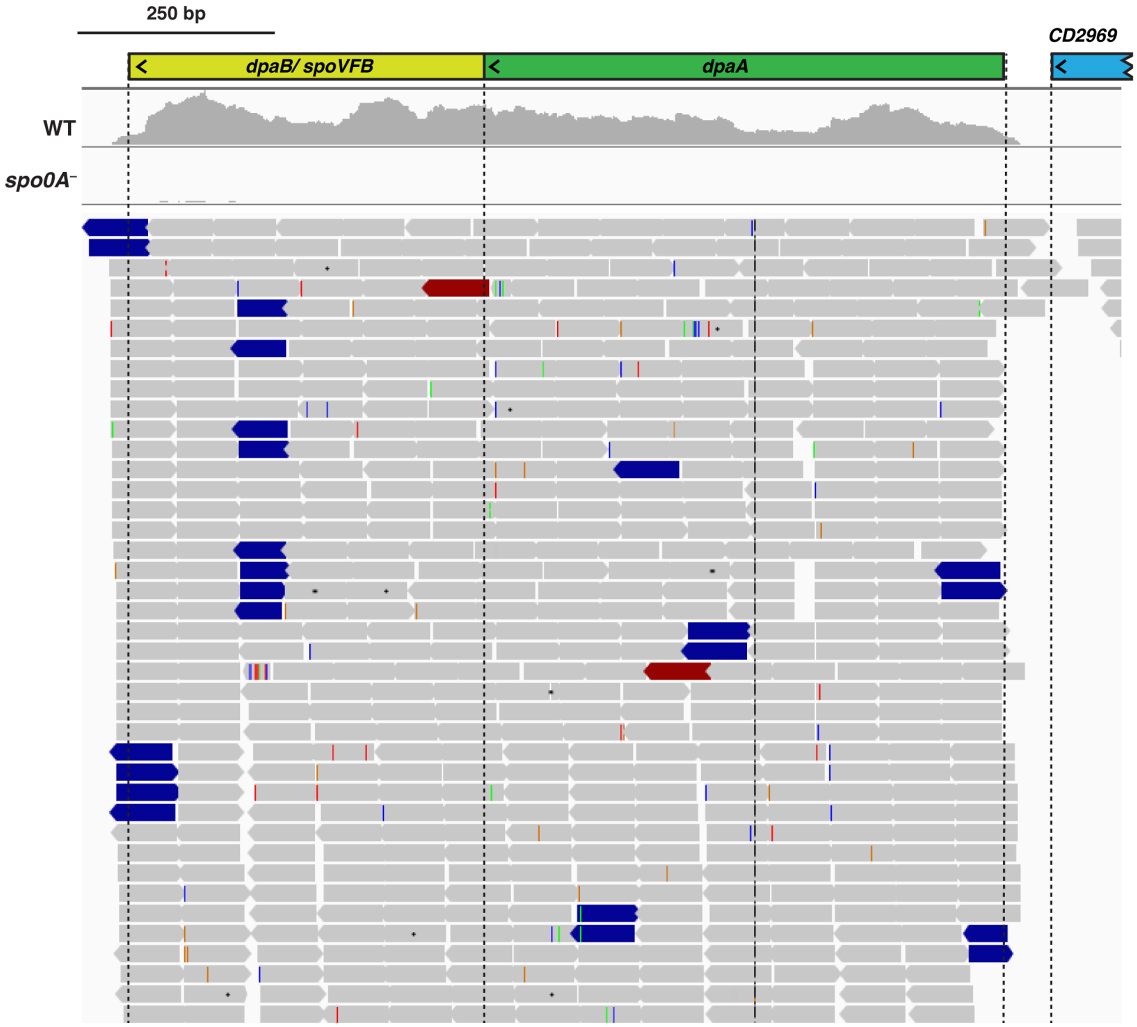

FIG S3. Representative image of Integrated Genome Viewer software (52) used to visualize of *dpaAB* RNA-Seq data from (40). Histograms of RNA sequence reads obtained in wild type and *spo0A*⁻ are shown in grey. The direction of transcription is indicated by the angled bracket. The reads mapped to this locus are shown.

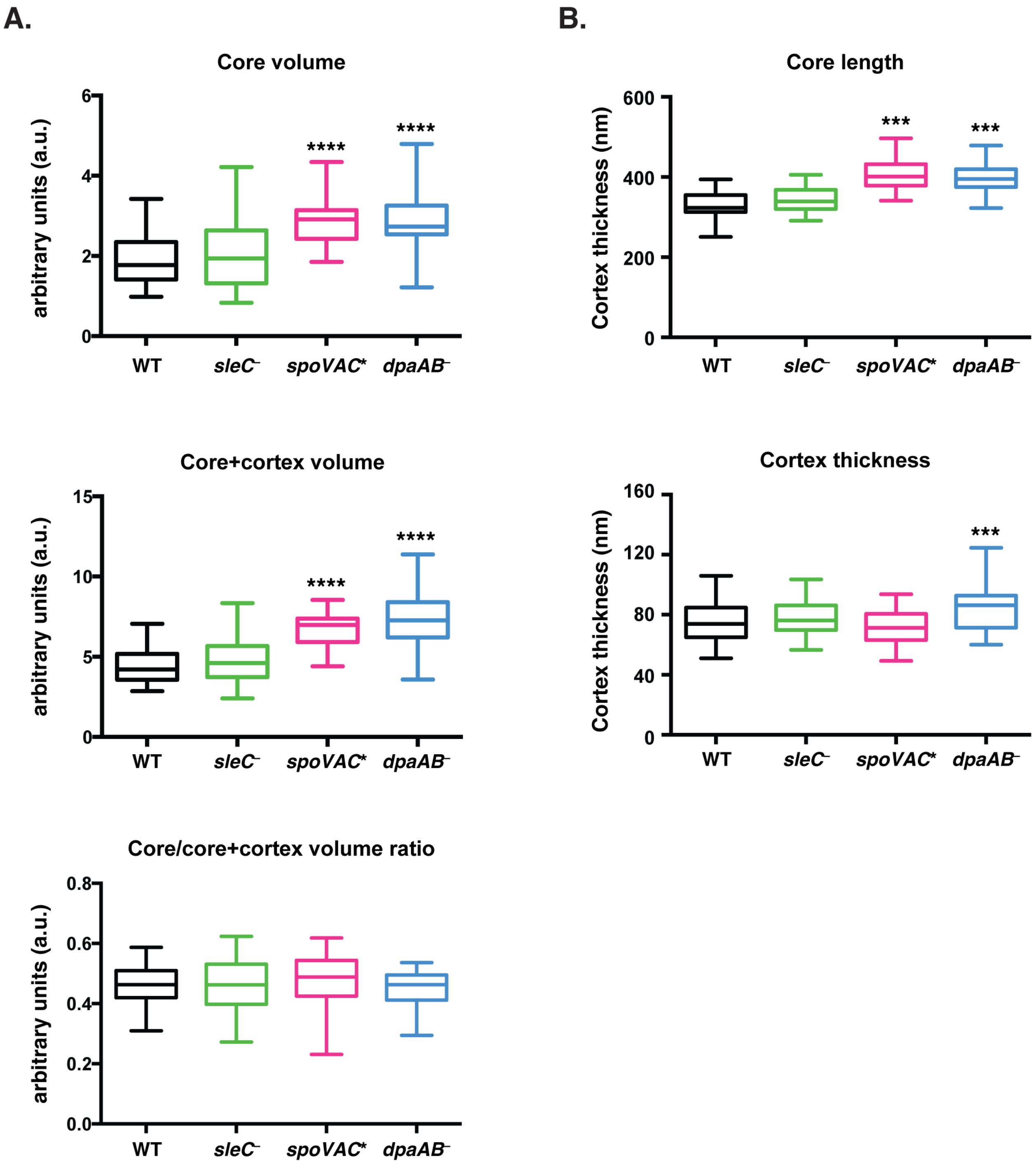

FIG S4. Transmission electron microscopy (TEM) analyses of wildtype, *sleC*⁻, *spoVAC**, and *dpaAB*⁻ spores. (A) Box and whiskers plot of core and core+cortex volumes determined from measurements of the average length and width of longitudinal sections of spores. The volume was estimated as described by Beaman *et al*. (44). These measurements derive from TEM

analyses of a minimum of 35 spores from the images used in Fig. 4. (B) Box and whiskers plot of cortex thickness and core length of *spoVAC** and *dpaAB*⁻ spores. Measurements derive from TEM analyses of a minimum of 50 spores from the images used in Fig. 4. Statistical significance was determined using ANOVA and Tukey's test (*** $p < 0.001$, **** $p < 0.0001$).

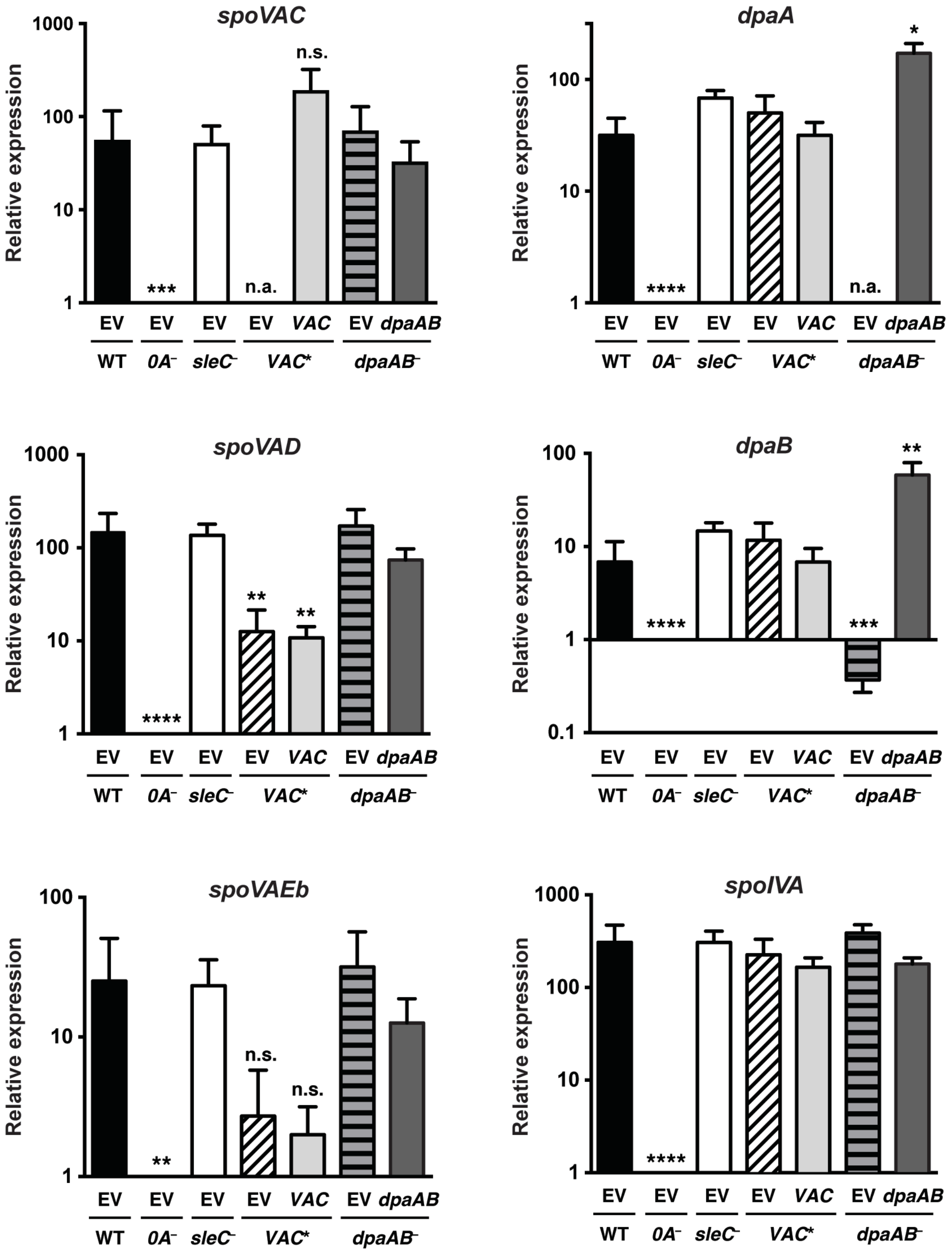

FIG S5. qRT-PCR analyses of *spoVA* and *dpaAB* transcript levels. RNA was isolated from sporulating cells of wildtype carrying empty vector (WT/EV), *spoVAC** or *dpaAB*⁻ carrying either empty vector or the indicated complementation constructs. Transcript levels were

normalized to the housekeeping gene *rpoB* using the standard curve method and are shown relative to the *spo0A*$^{-}$ strain. Data represents the average of three biological replicates. Error bars indicate the standard error of the mean. n.a. indicates not applicable, since the region amplified spans the disrupted gene.

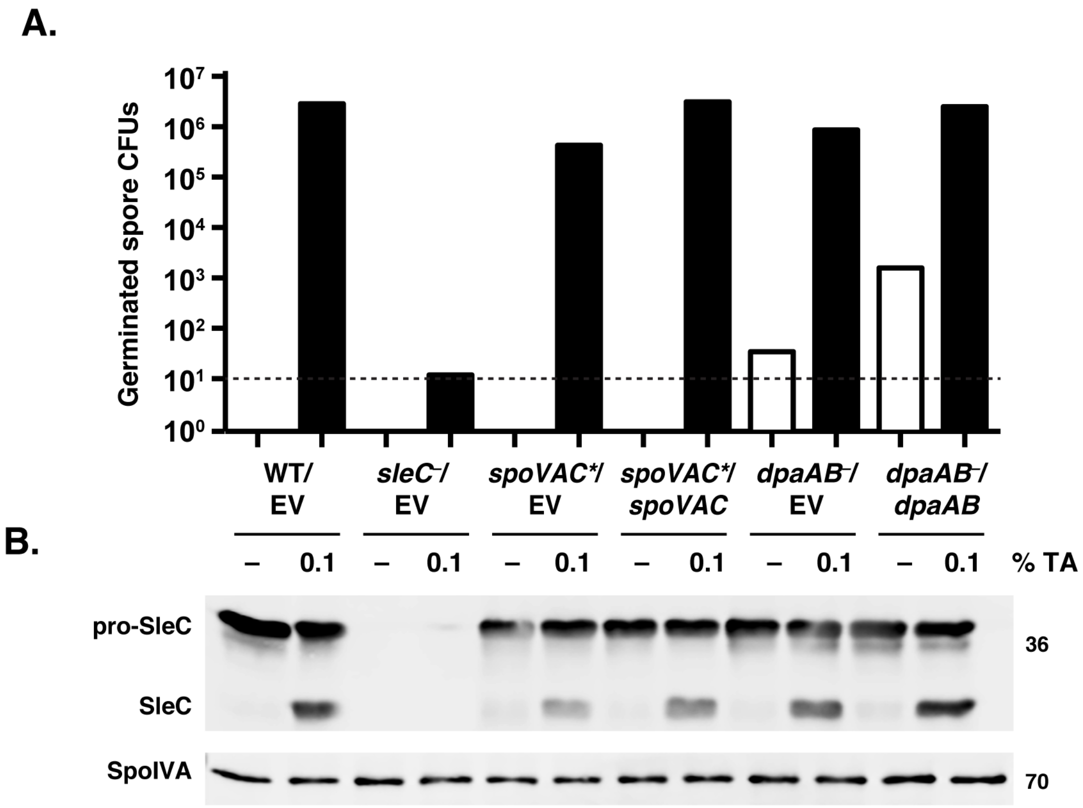

FIG S6. SleC cleavage and germination of untreated *spoVAC** and *dpaAB*⁻ spores. (A) Germination of indicated strains of purified spores in response to either 0% or 1% taurocholate exposure for 20 min. Samples were plated on BHIS to measure the number of spores that germinated during this incubation. Statistical significance was evaluated using ANOVA and Tukey's test (**** $p < 0.0001$). n.s. = no statistical significance. (B) Western blot analyses of samples from one representative replicate of the *in vitro* germination assay shown above. The zymogen pro-SleC is processed by CspB in response to taurocholate addition (12).

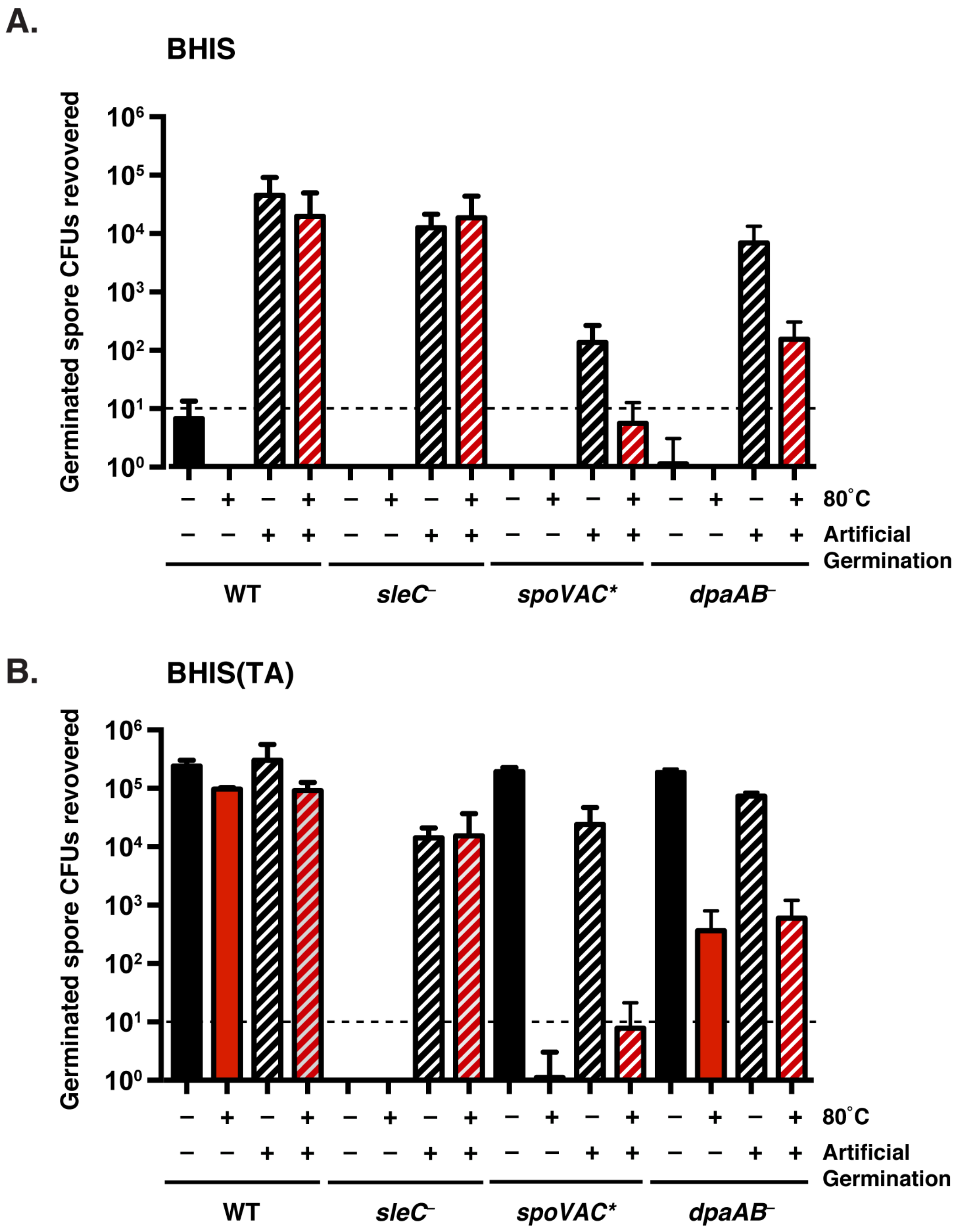

FIG S7. Artificial germination of either unheated or heat-treated wildtype, *spoVAC**, and *dpaAB*⁻ spores. Spores were either unheated or heated at 80˚C for 15 min prior to their incubation with thioglycollate and lysozyme (artificial germination) as previously described (13). The spores were serially diluted and plated on either (A) BHIS or (B) BHIS containing 0.1% taurocholate.

Untreated samples were exposed to the same heating and spinning procedures as the artificial germination samples. Data represents the average of 3 biological replicates.

Table S1. Primers used in this study.

Primer	Name	Sequence
532	3’ Universal EBS	CGAAATTAGAAACTTGCGTTCAGTAAAC
1714	5' IBS1 *spoVAC* 264	AAAAAAGCTTATAATTATCCTTAGGTTCCATAATTGTGCGCCCAGATAGGGTG
1715	3' EBS1d *spoVAC* 264	CAGATTGTACAAATGTGGTGATAACAGATAAGTCATAATTCCTAACTTACCTTTCTTTGT
1716	5' EBS2 *spoVAC* 264	TGAACGCAAGTTTCTAATTTCGATTGAACCTCGATAGAGGAAAGTGTCT
1735	5' *spoVAC* 22 bp	TATGTAGACCAAATAAGCCCAAAACC
1736	3' *spoVAC* 407 bp	CCACATAAAATAGAAGAACCTATCCC
1814	5' IBS1 *dpaA* 423	AAAAAAGCTTATAATTATCCTTAGCTATCGCAGAAGTGCGCCCAGATAGGGTG
1815	3' EBS1d *dpaA* 423	CAGATTGTACAAATGTGGTGATAACAGATAAGTCGCAGAAGCTAACTTACCTTTCTTTGT
1816	5' EBS2 *dpaA* 423	TGAACGCAAGTTTCTAATTTCGATTATAGCTCGATAGAGGAAAGTGTCT
1820	5' *dpaA* (22) for qRT	ACTGTTATTGGGGGAGACCTGC
1821	3' *dpaA* (151) for qRT	GGAACTTTTGGATAGTGCTTCAGC
1842	5' NdeI *dpaA*	AAAACATATGTCAAATCAATACGATATAA
1843	3' XhoI *dpaA* No Stop	AAAACTCGAGATCCCTTCTTTCATTTATAATATTTT
1855	5' NotI *spoVAC* upstream	AATGCGGCCGCGGAAAAATAGATTTTTTCTTTATTAGGC
1856	3' XhoI *spoVAC* +Stop Codon	AATCTCGAGCAATTAGTTACTTTAGTAGCTATTTTCAG
1891	5’ NotI *dpaA* 373 bp upstream	AATAGCGGCCGCGTGATGACATTATAAATATTGGAGG
1892	3’ XhoI *dpaB* over stop	AAGCTCGAGCTATTGATGTGGACTTTTTATAACAGGC
1931	5' *spoVAE* Start	ATGTTAATGGACTATGTAAGAGTATTTATTGTAG
1932	3' *spoVAE* Stop	TTATGGTTTTGCTTTTGGAGTAAATACTACTGAC
2030	5' *spoVAD* 471bp	TCAAAAACCAATGACAGCCCAG
2031	3' *spoVAD* 832bp	AACACCCACATCCACTTCCACC
2034	5' *spoVFB* 335bp	TGGCAGCAAAAGGTCATC
2035	3' *spoVFB* 386bp	GCTTAGAGCATCATTCGTTG

Restriction sites are underlined.

Table S2. A_{270} measurement of Total DPA in *spoVAC and *dpaAB⁻* spores**

	WT	*spoVAC⁻*	*dpaA⁻*	*sleC⁻*
Untreated Biol. Rep 1.1	0.018	0.199	1.360	0.013
Untreated Biol. Rep 1.2	0.018	0.398	1.200	0.013
Untreated Biol. Rep 1.3	0.023	0.429	1.205	0.015
Untreated Biol. Rep 2.1	0.009	0.064	0.051	0.071
Untreated Biol. Rep 2.2	0.009	0.010	0.008	0.018
Boiled Biol. Rep 1.1	0.202	0.253	1.420	0.205
Boiled Biol. Rep 1.2	0.181	0.477	1.299	0.177
Boiled Biol. Rep 1.3	0.176	0.471	1.260	0.186
Boiled Biol. Rep 2.1	0.287	0.196	0.135	0.347
Boiled Biol. Rep 1.1	0.293	0.105	0.089	0.322
ΔA_{270} Biol. Rep 1.1	0.184	0.054	0.060	0.192
ΔA_{270} Biol. Rep 1.2	0.163	0.079	0.099	0.164
ΔA_{270} Biol. Rep 1.3	0.153	0.042	0.055	0.171
ΔA_{270} Biol. Rep 2.1	0.278	0.132	0.084	0.276
ΔA_{270} Biol. Rep 1.1	0.284	0.095	0.081	0.304

Data represents the A_{270} values measured for three Biological Replicate (Biol. Rep) for spore preparation 1.X (spores were ~two months old) and two biological replicates of spore preparation 2.X (spores were one week old).
